# Deciphering T-cell exhaustion in the tumor microenvironment: paving the way for innovative solid tumor therapies

**DOI:** 10.3389/fimmu.2025.1548234

**Published:** 2025-04-01

**Authors:** Reshmi Nair, Veena Somasundaram, Anshu Kuriakose, Shiv Ram Krishn, David Raben, Rachel Salazar, Pradip Nair

**Affiliations:** ^1^ Syngene International Limited, Bengaluru, India; ^2^ Bicara Therapeutics, Boston, MA, United States

**Keywords:** T-cell exhaustion, T-cell activity, solid tumor, novel therapeutic approach, tumor microenvironment

## Abstract

In solid tumors, the tumor microenvironment (TME) is a complex mix of tumor, immune, stromal cells, fibroblasts, and the extracellular matrix. Cytotoxic T lymphocytes (CTLs) constitute a fraction of immune cells that may infiltrate into the TME. The primary function of these T-cells is to detect and eliminate tumor cells. However, due to the immunosuppressive factors present in the TME primarily mediated by Myeloid-Derived Suppressor Cells (MDSCs), Tumor associated macrophages (TAMs), Cancer Associated Fibroblasts (CAFs) as well as the tumor cells themselves, T-cells fail to differentiate into effector cells or become dysfunctional and are unable to eliminate the tumor. In addition, chronic antigen stimulation within the TME also leads to a phenomenon, first identified in chronic lymphocytic choriomeningitis virus (LCMV) infection in mice, where the T-cells become exhausted and lose their effector functions. Exhausted T-cells (Tex) are characterized by the presence of remarkably conserved inhibitory receptors, transcription and signaling factors and the downregulation of key effector molecules. Tex cells have been identified in various malignancies, including melanoma, colorectal and hepatocellular cancers. Recent studies have indicated novel strategies to reverse T-cell exhaustion. These include checkpoint inhibitor blockade targeting programmed cell death protein 1 (PD-1), T-cell immunoglobulin and mucin-domain containing-3 (Tim-3), cytotoxic T-lymphocyte associated protein 4 (CTLA-4), or combinations of different immune checkpoint therapies (ICTs) or combination of ICTs with cytokine co-stimulation. In this review, we discuss aspects of T-cell dysfunction within the TME with a focus on T-cell exhaustion. We believe that gaining insight into the mechanisms of T-cell exhaustion within the TME of human solid tumors will pave the way for developing therapeutic strategies to target and potentially re-invigorate exhausted T-cells in cancer.

## 1 Introduction

The TME of solid tumors is a very heterogeneous mix of tumor and other cell types (1–3). “Hot tumors” are characterized by the presence of immune cell such as T, B-lymphocytes, natural killer cells (NK), and dendritic cells (DC) within the TME.” (4). These immune cells are actively engaged in recognizing and attacking tumor cells and recent literature suggests that these are the tumors where ICT has maximum clinical benefit. In “cold tumors”, on the other hand, the TME is characterized by sparse infiltration or complete absence of immune cells and patients presenting with such tumors have poor prognosis. Between the “hot” and “cold” extremes of tumor types are a series of intermediate tumors which are at different degrees of “warmth” categorized by the numbers and types of immune cells within the TME (5).

Patients presenting with tumors showing presence of cytotoxic CD8^+^ T-cells within the TME have the best prognosis with ICT (6). To understand the dynamics of the presence or absence of these CTLs within the TME of solid tumors, it is important to understand the development path of T-cells. The origin of T-cells can be traced back to the bone marrow, where hematopoietic stem cells (HSCs) give rise to lymphoid progenitor cells (LPCs) (7). These LPCs then migrate to the thymus and differentiate into thymocytes, a type of immature T-cell. In the thymus, thymocytes undergo variable–diversity–joining (VDJ) recombination, leading to T-cell receptor (TCR) diversity, and positive or negative selection based on their ability to recognize self-antigens presented by Thymic Epithelial Cells (TECs) in the context of the Major Histocompatibility Complex (MHC) (8). High affinity thymocytes recognizing self-antigen-MHC complexes are eliminated in the thymus, leading to tolerance of T-cells to self-antigens. This mechanism of generating tolerance to self-antigens is critical for the prevention of autoimmune diseases (9). The next step of T-cell maturation is the differentiation of thymocytes to either a CD4^+^ or CD8^+^ T-cell lineage. Post maturation, CD4^+^ and CD8^+^ T-cells leave the thymus and populate the peripheral lymphoid organs. The primary role of these mature T-cells is to respond to foreign antigens presented on MHC by specialized cells called antigen presenting cells (APCs) (Example: dendritic cells). The CD4^+^ cells also called T helper (Th) cells because they “help” B cells make antibodies, are activated by antigens presented by MHC class II (MHCII) molecules, while CD8^+^ cells are activated by antigens presented by MHC class 1 (MHCI) molecules. The differentiation of CD4 and CD8 cells into effector cells is influenced by various factors including the strength of TCR binding, co-stimulatory molecule interactions and the cytokine milieu. For example, CD4^+^ T-cells achieve different phenotypes such as Th1, Th2, Th17 or regulatory T-cells (Tregs), each with unique effector functions (10) while CD8^+^ cells primarily differentiate into CTLs capable of killing target cells expressing antigen to which it was primed. Endogenous antigens derived from proteins degraded in the proteasome, transported through the endoplasmic reticulum (ER) and presented on the cell surface by MHCI molecules activate CD8^+^ cells (11). This pathway is crucial for immune responses against viruses, and other intracellular pathogens as well as for tumor surveillance. MHCII presents exogenous antigens that are typically taken up by APCs, macrophages and B cells after the antigens enter these cells by endocytosis or phagocytosis. The antigens that enter endosomes or lysosomes are then degraded into peptides and bind to MHCII molecules synthesized in the ER and are transported by endosomes and subsequently presented on the cell surface where they interact with CD4^+^ cells. The MHCII response pathway is important for initiating immune responses against extracellular pathogens (12) This immune response cascade begins with T-cell proliferation, following which a small subset (~5%) of these T-cells differentiate into memory cells while the majority die via apoptosis (10, 13, 14).

T-cells are important in immunosurveillance where the immune system recognizes and eliminates cancer and viral infected cells in the body (15). CTLs play an important role in this process as they can detect and destroy cancer cells through antigen-specific interactions with antigen primed CD8^+^ T-cells, as described previously. An essential aspect of tumor surveillance is the recognition of neo-antigens which arise due to mutations in cancer cells leading to the development of abnormal proteins which are not present in normal cells, thus making these cells amenable to immune detection. Other reasons for immune detection include TAAs (tumor associated antigens) altered by post translational modifications such as phosphorylation, glycosylation, methylation, acetylation, ubiquitination which generate neoantigens that enhance the immunogenicity of cancer cells by providing unique targets for immune cell recognition (16). This hypothesis suggests that modifying the proteins involved in antigen presentation machinery could alter the range of antigens presented to the immune system, thereby changing how the immune system recognizes cancer cells (16).

However, the process of immune monitoring is not absolute, and tumors often escape immunosurveillance. A major reason for escaping from immune detection by some tumors is attributed to the self-antigen presented on MHC by APCs within the TME and at local lymph nodes. T-cells that have been educated in the thymus, tolerate self-antigen, and thus fail to mount a substantial immune response (17). In other instances, the TME can generate an immunosuppressive environment mediated by TAMs, MDSCs, CAFs and even by the tumors themselves (18). Such an immunosuppressive environment can be induced by (a) suppressive cytokines such as interleukin 10 (IL-10) and transforming growth factor-beta (TGF-β) produced by innate cells, and Tregs present within the TME, (b) inhibitory receptors such as PD-1, CTLA-4, Tim-3, Lymphocyte-activation gene 3 (LAG-3), T cell immunoreceptor with Ig and Immunoreceptor Tyrosine-based Inhibitory Motif (ITIM) domains (TIGIT) overexpressed on immune cells and their corresponding ligands upregulated on tumor cells and APCs, thereby suppressing T-cell activation (19), and (c) sub-optimal antigen presentation by APCs leading to chronic T-cell activation and subsequent exhaustion instead of acute activation that can have anti-tumor effects (20). Sears et al. (2021), characterized exhausted T-cells using three critical features- (i) suboptimal effector functionality, (ii) persistent expression of inhibitory receptors, and (iii). a transcriptional state different from that of functioning effector or memory T-cells (21).

## 2 Mechanisms of T-cell dysfunction

### 2.1 T-cell fates during infection/cancer

**Figure 1:**
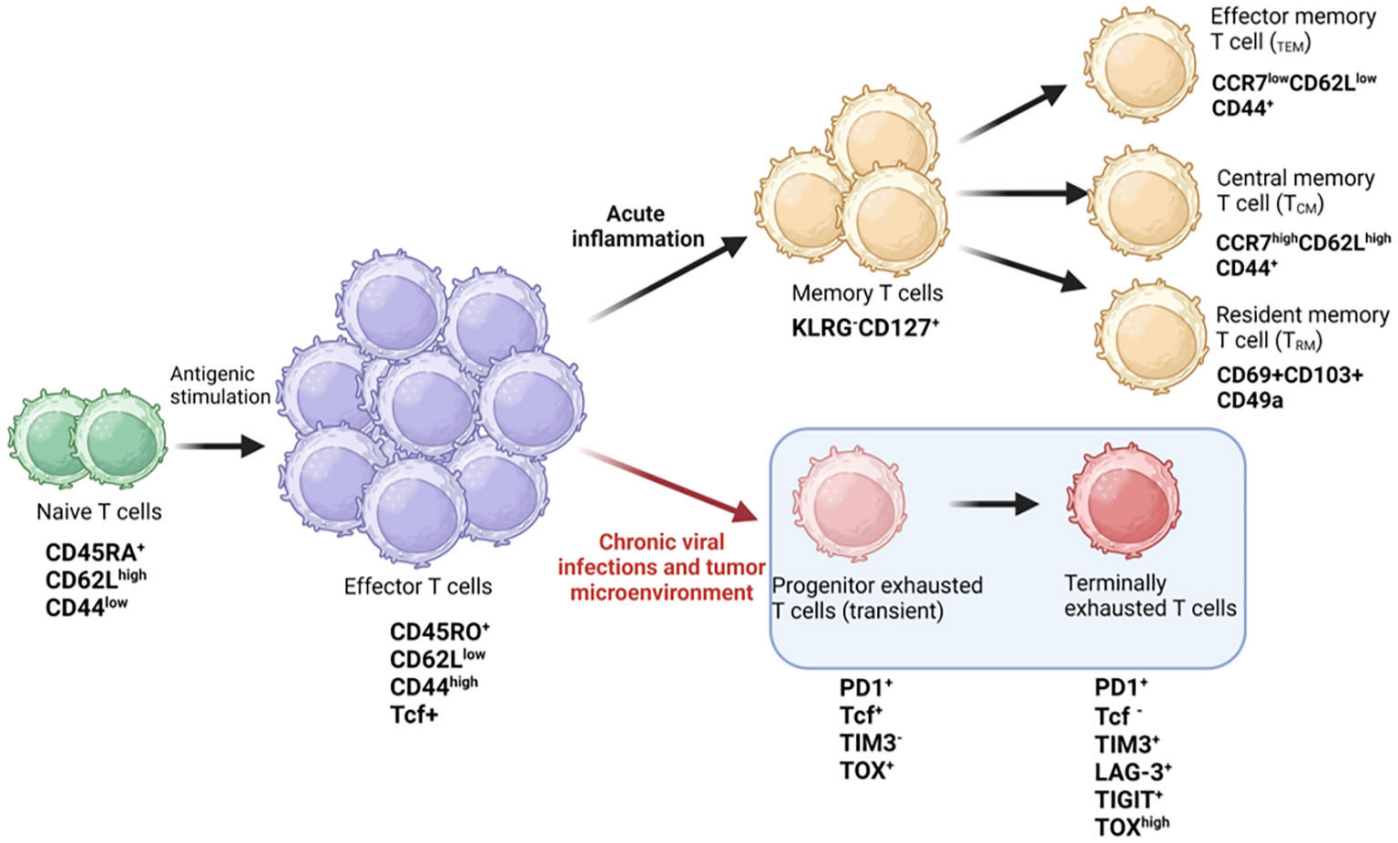
T cell fate during chronic infections. Diagrammatic representation showing antigenic stimulation of naïve T cells leading to signaling by TCR and co-stimulatory receptors, causing activation and proliferation of effector T cells generating immune response against pathogens. Antigen specific T cells differentiate into memory phenotypes, such as T effector memory (T_EM_), central memory (T_CM_), and resident memory T cells (T_RM_) for long-term immunity. During chronic viral infections or in the tumor microenvironment, the effector cells differentiate into progenitor (transient) exhausted T cells and finally into terminally exhausted or dysfunctional T cells. The expression of key cell surface receptors and transcription factors is depicted in the schematic and is derived from literature review. Figure prepared using BioRender.

Naïve T-cells (CD44^low^CD62L^high^), that have not encountered an antigen, are maintained in a state of quiescence at G0 stage of cell cycle, and are characterized by small cell size, low proliferative index, and reduced metabolism. Upon antigen recognition by the TCR-MHC complex (signal 1) followed by activation of costimulatory molecules such as CD28 complex (signal 2), T-cells are induced to leave the G0 phase. The G0 phase is also categorized as naïve or “immune innocent” stage. Activated T-cells (CD44^hi^CD62L^low^) increase in cell size, proliferation, and basal metabolism mediated by signal transduction downstream of the TCR complex resulting in activation of transcription factors, secretion of inflammatory cytokines and a battery of effector functions (22, 23). Activated T-cells are further characterized by upregulation of activation markers like CD25 and CD69, and an increase in the secretion of cytokines such as interleukin 2 (IL-2) and interferon gamma (IFN-γ) which enable the T-cells to proliferate and perform their effector functions (24). In normal physiology, once the infection is cured, the immune cells taper off through the elimination of 90-95% effector cells (CD44^hi^CD62L^low^) via apoptosis (14, 25, 26). The remaining T-cells convert to memory cells (T_M_), further classified as Teff memory (T_EM_), T central memory (T_CM_) and T resident memory (T_RM_) cells and are immediately mobilized in case of a re-infection (27). These long-lived memory T-cells confer protective immunity for extended periods (years) even after antigen withdrawal. However, during chronic infections or in a TME, the T-cells become hyporesponsive, decreasing their effector functions and hampering T-cell-mediated cytotoxicity of tumor cells. These hyporesponsive T-cells differentiate to progenitor exhausted T-cells (pTex) and terminally exhausted T-cells, ultimately becoming completely dysfunctional (Tex) (28, 29) (**Figure 1**).

### 2.2 T-cell anergy and senescence

**Figure 2:**
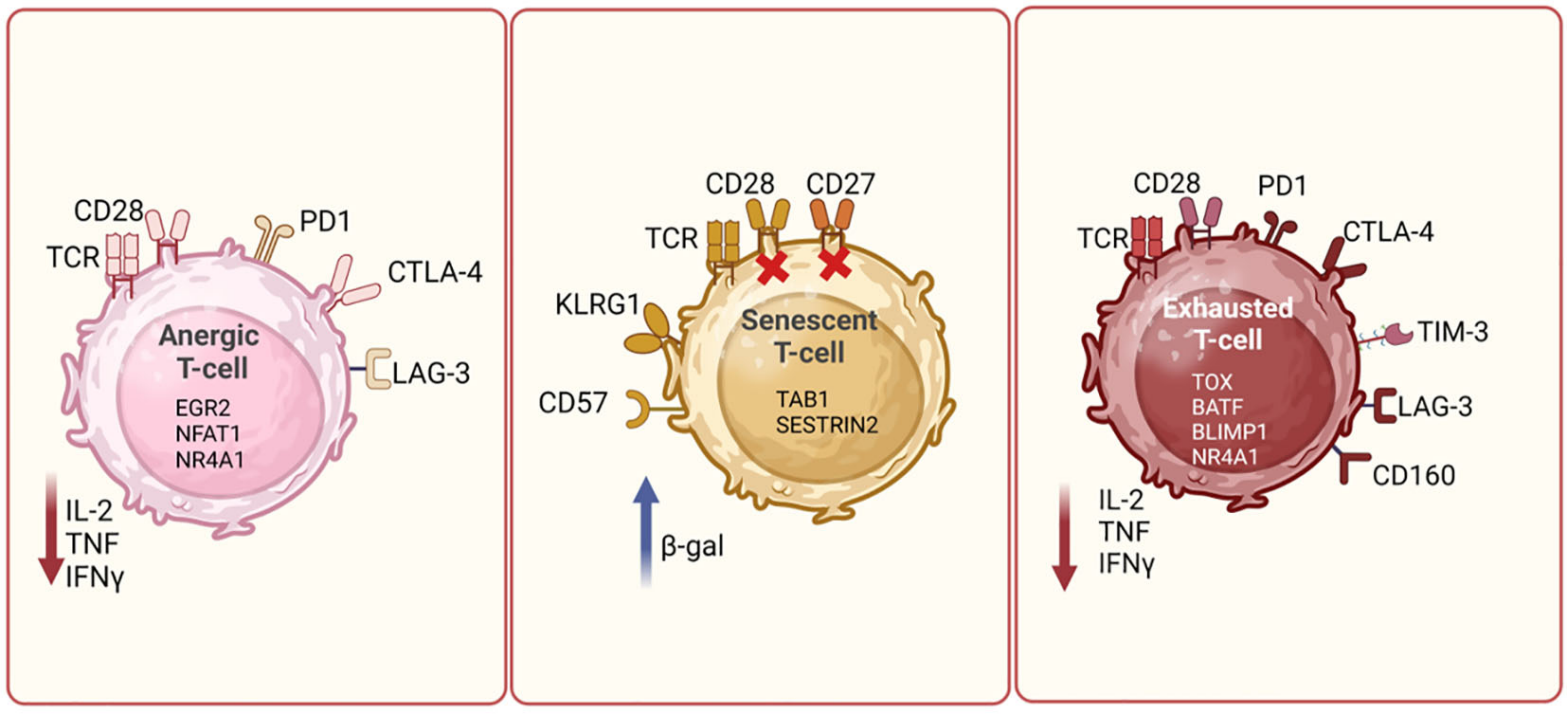
T cell dysfunction mechanisms. Diagrammatic representation showing differences between different types of dysfunctional T cells, such as exhausted, anergic and senescent T cells. Anergic T cells can be characterized by high expression of PD1, CTLA4 and LAG3, and reduced production of cytokines such as IL-2, TNF and IFN-γ. Senescent T cells are identified by the loss of CD28 and increased expression of CD57 and KLRG1. These cells often express β-galactosidase activity. Exhausted T cells are marked by the upregulation of PD-1, CTLA-4, TIM-3, and LAG-3. These cells exhibit reduced effector functions, including decreased cytokine production and cytotoxic activity. The expression of key cell surface receptors, transcription factors and cytokines secreted by these cells is depicted in the schematic and is derived based on literature review. Figure prepared using BioRender.

Anergy, senescence and exhaustion are three different mechanisms for hypo-responsiveness by T-cells (30–32). The mechanisms of anergy and senescence of T-cells have been well described in several publications. In an anergic state following antigen exposure, T-cells become hyporesponsive either due to sub-optimal co-stimulation by CD28 or an increase in co-inhibitory signaling mediated by receptors such as PD-1, LAG-3 and TIGIT (33). During anergy, T-cells typically exhibit decreased IL-2 production, impaired proliferation, and are metabolically inert (34). Impaired T-cell proliferation or anergy is mediated by various transcription factors such as Nuclear factor of activated T-cells-1 (NFAT1) (in the absence of AP-1), Early growth response protein-2 (EGR2) and EGR3, which regulate gene expression and TCR signaling (32, 35–37). Anergic T-cells are unresponsive or passive to subsequent activations and remain alive for extended periods (36, 38). Therefore, T-cell anergy has important implications in immune tolerance and autoimmunity, and tumor cells can also exploit T-cell anergy as an escape mechanism to evade detection and destruction by the immune system (32). On the other hand, senescent T-cells are terminally differentiated cells with defective TCR signaling. Apart from the secretion of tumor necrosis factor alpha (TNFα), osteopontin and senescence-associated secretory phenotype (SASP), these cells also have low telomerase activity, show signs of DNA damage, cell cycle arrest, apoptosis resistance and high beta-galactosidase activity. They often lose the expression of costimulatory molecules like CD27 and CD28 and upregulate the coinhibitory molecule, killer cell lectin-like receptor subfamily G member 1 (KLRG1) and CD57, a marker for replicative senescence of T-cells (32, 39). Recent reports have highlighted the roles for TAB1 and Sestrin proteins in T-cell senescence. AMP-activated protein kinase (AMPK), can trigger p38 auto-phosphorylation via the scaffold protein TAB1, leading to inhibition of T-cell proliferation and activation of telomerase activity on senescent T-cells (40). Similarly, sestrins, as stress-sensing proteins, have been implicated in blocking TCR signaling in CD27^-^CD28^-^CD8^+^ senescent T-cells. Expression of NKG2D receptor can reprogram these senescent T-cells toward innate killing activity, making them functional again (41, 42). Therefore, mechanisms to rejuvenate senescent T-cells may improve treatment response in cancer. Pictorial representation of some of the key markers expressed on anergic and senescent T-cells is shown in **Figure 2**.

In this review, we focus on the mechanisms driving T-cell exhaustion and its role in the TME as discussed in upcoming sections. Detailed discussion of other mechanisms of T-cell dysfunction is beyond the scope of this review.

### 2.3 T-cell exhaustion

T-cell exhaustion is the stepwise or progressive loss of T-effector (Teff) cell functions. The severity of chronic infections, and the intensity and duration of antigenic stimulation are critical determinants of exhaustion (19, 32). Exhaustion occurs when T-cells are continuously activated at the sites of chronic inflammation (32). Both CD8^+^ and CD4^+^ T-cells are amenable to loss of effector functions during chronic infections (31). T-cell exhaustion was first identified upon analysis of CD8^+^ T-cells from LCMV infected mice. Chronic LCMV infection led to T-cell exhaustion evidenced by the reduced anti-viral effects of CD8^+^CD69^hi^CD44^hi^CD62L^low^ cells (43). Currently, several cell-surface molecules/co-inhibitory receptors and transcription factors have been identified as drivers of T-cell exhaustion.

Regulation of coinhibitory receptors during T-cell activation and exhaustion is crucial for immune regulation, both in chronic infections and in cancer immunotherapy. Following activation, T-cells also express coinhibitory receptors such as CTLA-4 and PD-1. These receptors act as brakes on T-cell responses, preventing overactivation of T-cells, and maintaining peripheral T-cell tolerance (44, 45). Upregulated soon after T-cell activation, CTLA-4 competes with CD28 to bind to CD80/CD86, expressed on APCs, and delivers inhibitory signals that dampen T-cell responses (45). On the other hand, PD-1 binds to its ligands, PD-L1 and PD-L2, on various cells, including APCs and tumor cells, leading to inhibition of TCR-mediated signaling and reduction of cytokine production and T-cell proliferation (46). In chronic infections (e.g., HIV, HCV) and cancer, multiple coinhibitory receptors, including PD-1, CTLA-4, LAG-3, TIM-3, and TIGIT are upregulated on Tex. Interactions of LAG-3, TIM-3 and TIGIT with their respective ligands on APCs (MHCII, Galectin-9 and CD155) are crucial to dampening T-cell responses and driving T-cell exhaustion or dysfunction (47). The expression of co-inhibitory receptors is dynamically regulated during immune response. Initially, T-cells upregulate these receptors to modulate the response and prevent excessive inflammation due to T-cell activation. But their persistent upregulation in chronic disease states leads to T-cell dysfunction.

Increased expression of specific transcription factors such as B lymphocyte-induced maturation protein-1 (Blimp-1), Nuclear factor of activated T-cells, cytoplasmic 1 (NFATc1), Basic leucine zipper transcription factor, ATF-like (BATF), and thymocyte selection-associated HMG box (TOX) and loss of T-cell factor 1 (TCF1) are now known to be associated with T-cell exhaustion (28, 48, 49). Reduced production of inflammatory cytokines such as IFN-γ, TNFα and IL-2 further characterize exhausted T-cells (50, 51). Hudson et al. reported initial differentiation of stem-like Tcf-1^+^ CD8^+^ T-cells into a transitory population of CD101^-^Tim3^+^ cells that later converts into exhausted CD101^+^ Tim3^+^ cells. While the transitory CD101^-^Tim3^+^ T-cells expressed CXCR3 and produced proinflammatory cytokines thus controlling LCMV infection, the exhausted T-cells had limited capability for division and cytokine production (52). Subsequent studies then indicated that this phenomenon was not limited to viral infections but is also associated with chronic bacterial and parasitic infections, and cancer. Within the TME of many different solid tumors, there is now evidence of compromised T-cell activity due to exhaustion (14, 50, 51) leading to collapse of anti-tumor immunity and development of immune tolerance and acquired resistance (53).

Recently, Beltra et al., have described a four-cell-stage developmental framework for exhausted T-cells (54). These stages involve two TCF1^+^progenitor subsets, one of which is tissue restricted and quiescent while the other, more accessible subset is in systemic circulation. The latter gradually loses TCF1 as it divides and converts to a third intermediary T exhausted (Tex) subset. This intermediate subset has the potential to revert to effector cells during PD1-PDL1 checkpoint axis blockade by ICT. The authors finally suggest that this intermediary subset ultimately converts into a fourth, terminally differentiated subset of Tex cells that are resistant to standard ICT. The authors have delineated the interplay between key transcriptional regulators such as TCF1, T-bet, and TOX in the regulation of these exhausted subsets. In summary, therapeutic targeting of T-cell exhaustion mechanisms can potentially reverse the suppressive tumor microenvironment making immunotherapy more durable and efficacious (54–56).

## 3 Antigenic determinants of T-cell exhaustion within the TME

In contrast to immunity toward infectious diseases, some tumor antigens are only weakly immunogenic and tumor-specific T-cells have low precursor frequencies and TCR affinity (14, 57). This dampened T-cell response, typically to tumor antigens, is driven by the elimination of high affinity T-cell clones to self-antigen during thymic development (58) as mentioned previously. The few effector cells which enter the TME are also regulated by the complex immunosuppressive network of cancer-, immune-, stromal cells and cytokines present within the TME.

To understand how these effector cells transform into exhausted cells within the TME, cues are available from viral studies. In a recent study by Osuch et al, to understand whether T-cell exhaustion phenotype in chronic HCV infection was related to the repertoire of viral immune epitopes, a large prospective cohort of chronic hepatitis C patients with viral sub genotype (1b) was analyzed. Exhausted T-cell phenotypes identified by expression of hallmark inhibitory receptors, PD-1 and TIM-3 (31) at a single cell level on both global CD8^+^ and HCV specific T-cells were associated with specific autologous viral epitope sequence (59). T-cell exhaustion has also been found to have an association with disease severity in SARS-CoV-2 patients (60). As more effector cells become exhausted, many persistent viral infections become difficult to clear. Fluorescence assisted cell sorting (FACS) analysis shows that in patients, especially those in the intensive care unit, levels of PD-1 and TIM-3 on both CD4^+^ and CD8^+^ are higher (60, 61). Serum from chronically ill patients showed high levels of IL-10, indicating a functionally suppressed T-cell phenotype (62). Interestingly, ICT has been explored in addition to anti-viral therapy (63, 64) in COVID patients with severe disease associated with exhausted T-cell phenotypes. In another study with head and neck derived tissues, CD8^+^ T cells specific to HPV proteins E2, E5 and E6 were identified by MHCI tetramers. These CD8^+^ T-cells expressed PD-1 and three different flavors of T-cells were identified. One subset expressed TCF7, and other genes associated with PD-1^+^ stem-like CD8 T-cells that are critical for maintaining T-cell responses in conditions of antigen persistence. The second subset expressed more effector molecules, representing a transitory cell population, and the third subset was characterized by a terminally differentiated gene signature. The authors suggest the use of ICT as a treatment option in these patients to boost their HPV specific T-cell response (65).

Therefore, within the TME, T-cells fates could be determined by TCR- epitope-MHC interaction leading to a more chronic T-cell activation pathway subsequently driving T-cells into exhaustion. Experiments involving co-culture of Jurkat cells with HepG2 tumor cells showed that PD-1 expression on Jurkat cells increased after co-culture with cancer cells leading to cell cycle arrest of Jurkat cells, and blockade of PD-1 pathway by an anti-PD1 monoclonal antibody successfully restored T-cell function (66). Studies indicate that tumors co-opt certain immune-checkpoint pathways as a major mechanism of immune resistance, particularly against tumor antigen-specific T-cells (67, 68). Recently, non-mutated immunogenic epitopes have been identified in genes such as erythroblastic oncogene B2 (ERBB2) in breast and ovarian cancers and H4 histone in prostate cancer, suggesting that germline variants may play a role in immunosurveillance. In a recent study, leveraging metadata from over 5870 breast cancer lesions, the authors showed that individuals with high germline epitope burden in ERBB2 were less likely to develop HER2 positive breast cancer compared to other subtypes. However, tumors that overcame such immune mediated negative selection are more aggressive and demonstrate an “immune cold” phenotype (69). The data indicated that the germline genome plays a role in somatic evolution. Further, Shakiba et al. propose a critical “Goldilocks” range of TCR signal strength which allows tumor specific T-cells to maintain a cell intrinsic functional program and execute tumor effector functions *in vivo* (70). These T-cells with intermediate range of TCR signal strength exhibit effective anti-tumor responses while T-cells outside of this window are either functionally inert or dysfunctional, and exhausted. A study assessing the immunogenicity of over 500 predicted neoantigen derived peptides in lung adenocarcinoma and melanoma patients showed that the epitopes with the highest affinity were non-immunogenic and majority of the immunogenic peptides were found to have an intermediate affinity (71).

## 4 Rise of exhausted T-cells within the TME

Immunological conditions within the TME leading to evasion of immune-surveillance or response to ICT are driven by several factors and a variety of cell types. These factors can prevail at an organismal level (patient-specific) or be more localized to the TME (tumor-specific). Exhaustion of T-cells is also influenced by several intracellular and extracellular factors that are discussed in detail in the following sections.

### 4.1 Patient-specific determinants of tumor T-cell exhaustion

Gut microbiota (72) and patient age (39) are two important factors that modulate T-cell responses. Studies into these factors as determinants of immune responsiveness of a tumor have recently gained traction.

#### 4.1.1 The gut microbiome

The human gut hosts myriad microorganisms, such as bacteria, virus, and protozoa, forming a complex and symbiotic ecosystem (73). They play critical roles in maintaining metabolic balance, integrity of the mucosal barrier, and modulating host immune response. Fluctuations in gut microflora during infections and cancer have been an area of extensive research in the last decade. Recent studies suggest that specific microbes influence T-cell differentiation, and an imbalance can promote a state of chronic inflammation leading to T-cell exhaustion. The responsiveness of metastatic melanoma patients to Ipilimumab was based on the type and abundance of baseline gut microbiota, as high *Faecalibacterium* showed longer progression free survival (PFS) (74). Another study found that efficacy of anti-CTLA-4 treatment in mice and patients was determined by T-cell responses specific for the Bacteroides namely, *B. thetaiotaomicron* or *B. fragilis* (75). Recent analyses of European registries and consortia revealed that >50% of stage III and stage IV cancer patients have gut dysbiosis compared to 20% in healthy volunteers (76). Studies have investigated the correlation of gut dysbiosis with inflammatory and malignant diseases of the GI tract. It has also been found that gut dysbiosis impacts response to anti-PD-1 therapy in melanoma patients. A study found significant differences between the composition and diversity of the gut microbiome of responders versus non-responders in an anti-PD-1 clinical trial on melanoma patients. Higher alpha diversity and abundance of Ruminococcaceae family bacteria was found in the responders (77). Additionally, in clinical trials, fecal microbial transplantation (FMT) abrogated resistance to anti-PD-1 therapy in melanoma patients and made the treatment naïve patients more susceptible to anti-PD-1 therapy thus indicating that gut dysbiosis could cause T-cell exhaustion that is then reversed by FMT (76). A similar response to FMT was observed in non-small cell lung cancer patients treated with anti-PD1 therapy (78). FMT with *Akkermansia muciniphila* was also effective in improving immunotherapy response in several other epithelial tumors and this effect was attributed to an IL-12 dependent recruitment of CCR9^+^ CXCR3^+^ CD4^+^ T lymphocytes into the TME (79). Additionally, within the immune cells (T-cells), mTOR (mammalian target of rapamycin) and Signal transducer and activator of transcription 3 (STAT3) activation occurs (80) leading to IL-17, IL-12 and IFN-γ production which can in turn lead to activation and improved response to anti-PD-1 therapy. These studies indicate that the gut microbiota influence T-cell responses toward effector function versus exhaustion. Targeting this gut microbiota could potentially reverse T-cell exhaustion, restore immune competence, and optimize therapeutic response (74, 81–83).

#### 4.1.2 Aging

Another patient-specific factor, aging, is a multi-pronged mechanism that acts at the cellular and sub-cellular levels to manifest organismal changes. It leads to three critical changes that then subsequently trigger downstream effects. Metabolic dysfunction is the first key change. This can occur due to increased production of reactive oxygen and nitrogen species (ROS/RNS) leading to lack of redox balance, sub-optimal functioning of mitochondria, impaired fatty acid metabolism due to increased, age-associated adiposity (84). In the specific context of T-cells, naïve/quiescent T-cells are known to be more catabolic in nature, possessing energy efficient metabolic processes while activated T-cells use anabolic (84) processes for protein synthesis and proliferation through less energy efficient methods (85). This has a direct effect on T-cell proliferation and function because accumulation of ROS leads to impaired oxidative phosphorylation and increased glycolysis leading to rapid consumption of glucose and accumulation of lactate. The reduced availability of glucose impairs further T-cell proliferation while low pH due to high levels of lactate prevents T-cell activation, subsequently leading to formation of exhausted T-cells. The second important aging-related change is genomic instability both due to genetic changes (DNA damage repair deficiency, increased mutations) as well as epigenetic changes. As explained in a later section, epigenetic changes play a critical role in nudging the pre-exhausted T-cells toward terminal exhaustion. Lack of homeostasis between protein synthesis and degradation is another critical factor that can affect important enzymes and all cell types (85). Higher incidence of age-related inflammation can also hasten T-cell exhaustion. Another suppressive phenotype, namely senescent T-cells share several metabolic, epigenomic and cellular signaling features with T-cells isolated from older individuals (86–88). A recent study found that there was an overexpression of exhaustion markers, TIM-3 and PD-1 in T-cells from aged mice as evidenced by an accumulation of TIM3^+^/PD-1^+^ CD8^+^ T-cells (89).

### 4.2 Tumor-specific determinants of T-cell exhaustion

Tumor-specific determinants including tumor mutational burden (90), tumor immune phenotype (i.e. hot or cold tumor), and non-tumor cell types present in the TME (5, 91) are well known/well-studied determinants of tumor-infiltrating T-cell fates. It is the impaired infiltration of T-cells into the TME that makes a tumor cold, and subsequently unresponsive to therapy. To add to the conundrum, majority of these few TILs get rapidly exhausted due to the nature of the TME especially in solid tumors where the major components of TME are TAMs, CAFs and MDSCs, all of which are immune suppressive. The roles of each of these cell types in T-cell exhaustion will be discussed in later sections. Another tumor-specific feature that can determine T-cell fate is epigenetic modification of cells within the TME. These epigenetic changes leading to changes in gene expression or immune cell metabolism led to either immune evasive or responsive features within cells in the TME (92–95) and their effects on T-cell exhaustion are discussed in detail here.

Epigenetic changes that occur in T-cells within the TME play critical roles at all stages of the T-cell differentiation process including T-cell exhaustion. Epigenetic changes occur at the chromatin level to activate a set of transcription factors entirely different from the gene set that defines T-effector cells and can also concurrently induce metabolic changes that then lead to T-cell exhaustion and death. An example of this is the impairment of oxidative phosphorylation that is an important metabolic ‘mark’ that pushes effector T-cells into an exhausted phenotype. Chromatin features, mainly acetylation and methylation determine the functional state of T-cells. It was found that increased H3K4me3, H3K9ac, H3K27ac and reduced H3K27me3 marks in a set of genes forms the ‘epigenetic signature’ of progenitor exhausted T-cells. A subsequent increase in H3K27me3 mark in the same set of genes, along with activation of another set of genes, then marks a T-cell as an exhausted T-cell (95). These specific gene sets include signal transduction proteins as well as genes that regulate metabolism and cytokine production (95). Epigenetic control over gene expression, in fact drives the changes that occur when T-cells transition from effector to exhausted phenotypes. Terminally exhausted T-cells also possess a bivalent chromatin that contains both H3K4me3 (activation mark) and H3K27me3 (repression mark) but lacks gene transcription. Hypoxia (as observed in the tumor core) is a trigger that leads to an increase in bivalent chromatin (95). Cumulative increase in DNA methylation programs driven by DNA Methyltransferase 3 Alpha (DNMT3A) can limit effector T-cell response and cause the T-cells to progress to exhaustion. Knockout of DNMT3A in precursor exhausted CD8^+^ T-cells led to the development of T-cells with the ability to respond to ICT even upon chronic antigen stimulation (96). Over-expression of Histone demethylases also has been shown to reactivate T-cells and induce antitumor response, similar to the effects observed with the use of HDAC (histone deacetylases) inhibitors (97, 98). HDAC inhibitors increased gene expression leading to CD8^+^ T-cell function while also reducing the expression of chemo attractants of MDSCs (99). Use of HDAC inhibitor along with PD1 blockade, significantly improved the proliferation of tumor infiltrating CD4^+^ and CD8^+^ T-cells (100). Self-renewal capacity of T-cells is essential to maintain T-cell plasticity and permit response to antigen exposure/immunotherapy. A critical transcription factor that regulates the conversion of pre-exhausted T-cells to a fully exhausted state is TCF1, and when the T-cells no longer express TCF-1, BATF or c-Jun and start expressing markers such as NFATc1, Interferon Regulatory Factor 4 (IRF4), TOX, they proceed toward terminal exhaustion (101). Lysine-specific demethylase 1A (LSD1), enhancer of zeste homolog 2 (EZH2), histone-lysine N-methyltransferase SUV39H1 and Tet methylcytosine dioxygenase 2 (TET2) have been found to regulate T-cell differentiation potential and T-cell exhaustion through DNA methylation and histone modifications that alter gene expression patterns (102–105). A study that analyzed the effects of a panel of structurally diverse HDAC inhibitors found that the ability of HDAC inhibitors to induce effector cell function is reversed when the T-cells were cocultured with Tregs, wherein Tregs seem to be driving the exhaustion of the T-cells (98) thus alluding to the significance of the role played by the other cells in the TME, toward induction of T-cell exhaustion in addition to the epigenomic changes occurring within the T-cells.

It is however worth mentioning that while many novel therapeutics targeting these mechanisms have entered clinical development, there is still no clinical validation of the hypothesis that modulating these pathways could have a meaningful efficacious effect. In fact, ATAC-seq (Assay for transposase-accessible chromatin with sequencing) analysis of basal cell carcinoma samples (paired samples, pre- and post-anti-PD1 blockade therapy) showed that exhausted T-cells within the TME had a completely distinct epigenetic state compared to other T-cells (106). It was not simply an activation of inhibitory receptors on preexisting effector T-cells. It was found that complete epigenomic reprogramming occurs in T-cells with the exhausted T-cells expressing just as many regulatory features and transcription factors as other T-cell subtypes. Hence, blockade of these receptors would not cause a reversion to effector T-cell phenotype. This could probably explain why a terminally exhausted T-cell phenotype cannot be reversed to an effector phenotype (105, 107).

### 4.3 Extracellular factors influencing T-cell exhaustion

Metabolites that accumulate within TME can influence T-cell fate. For example, elevated extracellular levels of potassium, released by necrotic tumor cells into the extracellular fluid of tumor, metabolically reprograms T-cell to induce stemness, self-renewal of T-cells, impairs TCR-driven Akt–mTOR phosphorylation and effector programs (108, 109). Extracellular vesicles, that are bioactive packs of proteins, lipids, and nucleic acids, can also modulate the TME by interacting with adjacent cells and promoting exhaustion. In a murine model of hepatocellular carcinoma, miR-21-5p carried by M2 macrophage-derived extracellular vesicles were found to facilitate CD8^+^ T-cell exhaustion by targeting YOD1 and activating the YAP/β-catenin pathway (110). It has also been reported that TME rich in neutrophil extracellular traps (NETs) expresses multiple inhibitory receptors and induces exhaustive phenotypes in CD4^+^ and CD8^+^ T-cells (elaborated in a later section). In mouse models, blocking PD-L1 helped in diminishing tumors, reducing neutrophil infiltration and formation of NETs, and reinvigorating T-cells (111, 112). Additionally, tumor type can also influence exhaustion of T-cells, with severe exhaustion signatures being observed in infiltrating T-cells of gliomas as compared to other tumors (113). Other than these factors, abnormal mitochondrial dynamics, and extrinsic factors such as hypoxia can cause metabolic, transcriptomic, and epigenetic damage to T-cells, resulting in T-cell exhaustion and dysfunction in the TME. In this regard, dynamin-related protein 1 (Drp1) affects T-cell function by mediating mitochondrial fission and maintaining dynamic mitochondrial networks (114). Rapidly growing tumor cells consume glucose and release lactate which accumulates and impedes T-cell growth thus forcing these T-cells into a hypo-responsive state.

### 4.4 Intracellular factors driving T-cell exhaustion

Intrinsic to the cells, TCF-1^+^ stem-like T-cells, show overlapping features with memory CD8^+^T-cells and Tex. Transcription factors such as BACH2 and MYB maintain progenitor exhausted T differentiation, stem-like properties, and prevent terminal exhaustion of T-cells (115, 116). Conversely, downstream of NFAT, activation of transcription factors such as TOX and NR4A, drives exhaustion in CD8^+^T-cells (117). Hence, the transcriptional interplay among NFAT, TOX, and NR4A protein families influences T-cell exhaustion and inhibition of these factors that promote T-cell exhaustion could have a role in improving effector functions in cancer immunotherapy.

Metabolic factors also influence intracellular functions in CD8^+^T-cells and drive them toward a state of exhaustion and dysfunction. Hung et al. reported a significant finding on the recycling of methionine, leading to elevated levels of 5-methylthioadenosine (MTA) and S-adenosylmethionine (SAM). This metabolic shift correlates with T-cell exhaustion in hepatocellular carcinoma (HCC) (118). Additionally, the deletion of Methionine Adenosyltransferase 2A (MAT2A), a pivotal enzyme in SAM production, demonstrated the inhibition of T-cell dysfunction and suppression of HCC growth in murine models. These findings highlight the potential utility of serum MTA levels as an indicator for exhaustion and overall survival in HCC patients (118). Interestingly, mitochondrial dysfunction can drive the transition from precursor to terminally exhausted T-cells. Mitochondria-induced redox stress impedes the proteasomal degradation of hypoxia-inducible factor 1α (HIF-1α), thereby promoting transcriptional and metabolic reprogramming of cells ranging from precursor to terminally exhausted T-cells (119). Recent studies have reported a link between TIGIT and hypoxia inducible factor 1-α (HIF1-α). TIGIT impacts adenosine receptor signaling in T-cells, interferes with T-cell energy metabolism, and is associated with the kynurenine pathway in tumor cells, thereby altering the TME and T-cell-mediated immunity against tumors (120).

This re-emphasizes the importance of the role of metabolic reprogramming in determining T-cell fate within the TME. An understanding of these diverse intracellular and extracellular factors provides a comprehensive view of T-cell exhaustion and highlights potential therapeutic targets for enhancing immune responses against chronic infections and cancers.

## 5 Cellular factors within TME associated with T-cell exhaustion

### 5.1 Cancer-associated fibroblasts and T-cell exhaustion in the TME

CAFs are critical mesenchymal cells present within the TME that are also the most prominent non-neoplastic cell type (121, 122). Primarily thought to differentiate from tissue-resident fibroblasts, CAFs can also be generated from endothelial cells, pericytes, circulating mesenchymal cells as well as adipocytes (122). CAFs are involved in ECM remodeling in injury and in cancer. These cells are famously heterogenous, making them elusive therapeutic targets. In most cancer types a prominence of CAFs has been associated with poor prognosis except in Pancreatic Ductal Adenocarcinoma (PDAC) where the presence of Fibroblast activation protein (FAP^+^) and alpha smooth muscle actin (α-SMA^+^) cells associated with increased survival (123, 124). Depletion of FAP^+^ CAFs in mammary tumor models has shown a shift from IL-4 and IL-6 expression to IL-2 and IL-7 expression in tumor homogenates. This indicates that CAFs drive an immune suppressive Th2 response (122). Similarly, α-SMA^+^ CAFs have been found to promote mammary tumor growth by fueling cancer cell metabolism directly (125). Hence, CAFs have varied mechanisms by which a pro-tumor microenvironment is maintained. CAFs can be the gatekeepers that prevent entry of TILs into the tumor niche (immune exclusion). These CAFs can also cause direct or indirect modulation of T-cell activity including the release of chemokines such as CXCL12 that attract the CD8^+^ T-cells to themselves and keep them away from the tumor (122). An interesting study found that CAF-derived conditioned media led to increased tumor cell PD-L1 expression via CXCL2 in lung cancer cells and via CXCL5 in murine melanoma and CRC. Conditioned media also significantly reduced the expression of CD69 (early activation marker) on CD8^+^ T-cells and dampened the production of granzyme B (122).

Recent studies allude to the different CAF subsets with a range of immune-modulatory functions across cancer types (126). Cancer-associated fibroblasts can be directly involved in T-cell suppression/exhaustion or can have indirect effects via their interactions with different types of innate cells as well as NK cells (126) which in turn modulate T-cell activity within the TME. Recently, three broad subtypes of CAFs have been identified (127) called myofibroblast-like RGS5+ CAFs (myCAFs), matrix CAFs (mCAFs), and immunomodulatory CAFs (iCAFs). TGFβ-associated myCAFs correlate with increased Treg recruitment and differentiation, and enhanced expression of PD-1 and CTLA4 (122). In low-grade tumors, mCAFs produce the extra cellular matrix and prevent T-cell entry into the tumor cell vicinity while iCAFs operate within high-grade tumors to produce cytokines and chemokines for immune infiltration and modulation (127).

Reflecting the heterogeneity of CAFs, recent studies have identified several markers expressed on CAFs that may be unique to each cancer type. The expression of several of these markers are associated with features of T-cell exhaustion/inhibition (**Table 1**).

**Table 1:** Summary of the different CAF markers that have been identified in recent years across different cancer types and their effects on T-cell activity/exhaustion as well as its effects on prognosis (where available).

Cancer type	CAF Marker	Association of CAF marker expression with T cells	Reference
Lung adenocarcinoma	COL11A1	COL11A1 overexpression leads to poor prognosis, Positive correlation with expression of HAVCR2, CD274, CTLA4, LAG3 in T cells	(128)
Pancreatic ductal adenocarcinoma	TAK1	TAK1 inhibition leads to high CD4^+^ and CD8^+^ T-cell numbers; reduced immunosuppressive cells	(129)
Bladder cancer	Activated JNK signaling	Inhibition of JNK signaling causes downregulation of TSLP1; restoration of CD8^+^ T-cell effector function	(130)
Colorectal carcinoma (CRC)	CD40 and NECTIN2	CD40 and NECTIN2 blockade significantly reduced the expression of CD25, TIM-3, LAG3, and PD1; poor patient prognosis	(131)
Hepatocellular carcinoma	CD36	CD36 inhibition leads to a reduction in IL-6, TGF-β and VEGF-α; reduced expression of PD-1 on CTLs	(132)
CRC	α2,3/6-linked sialic acid, Sialyltransferase, Siglec ligands	Overexpression of these markers drives an increase of CD8^+^ PD1^+^ and CD8^+^ Siglec-7^+^/Siglec-9^+^ T cells	(133)

CAFs also drive T-cell exhaustion indirectly via interactions with innate cells and subsequent cytokine/chemokine production. CAFs can recruit neutrophils, monocytes and macrophages and drive the formation of M2 macrophages. Chitinase-3 like protein-1 derived from CAFs in addition to recruiting macrophages and converting them into tumor associated macrophages, also stimulate a Th2 response and prevents CD8^+^ T-cell infiltration (126). Through the production of PGE2 and IDO, CAFs impede NK cell proliferation in hepatocellular carcinoma. Dendritic cell maturation and antigen presentation capabilities are also affected by CAFs (126). The innate cells thus affected by CAFs subsequently prevent T-cell activation or induce T-cell exhaustion as described in the upcoming section.

### 5.2 Innate cell induced T-cell exhaustion

Tumor cells, under conditions of hypoxia and acidic environment can release chemokines and cytokines that then affect the innate cells such as neutrophils, dendritic cells, tumor associated macrophages (TAMs/M2) as well as MDSCs to drive T-cell exhaustion/inhibition.

**Figure 3:**
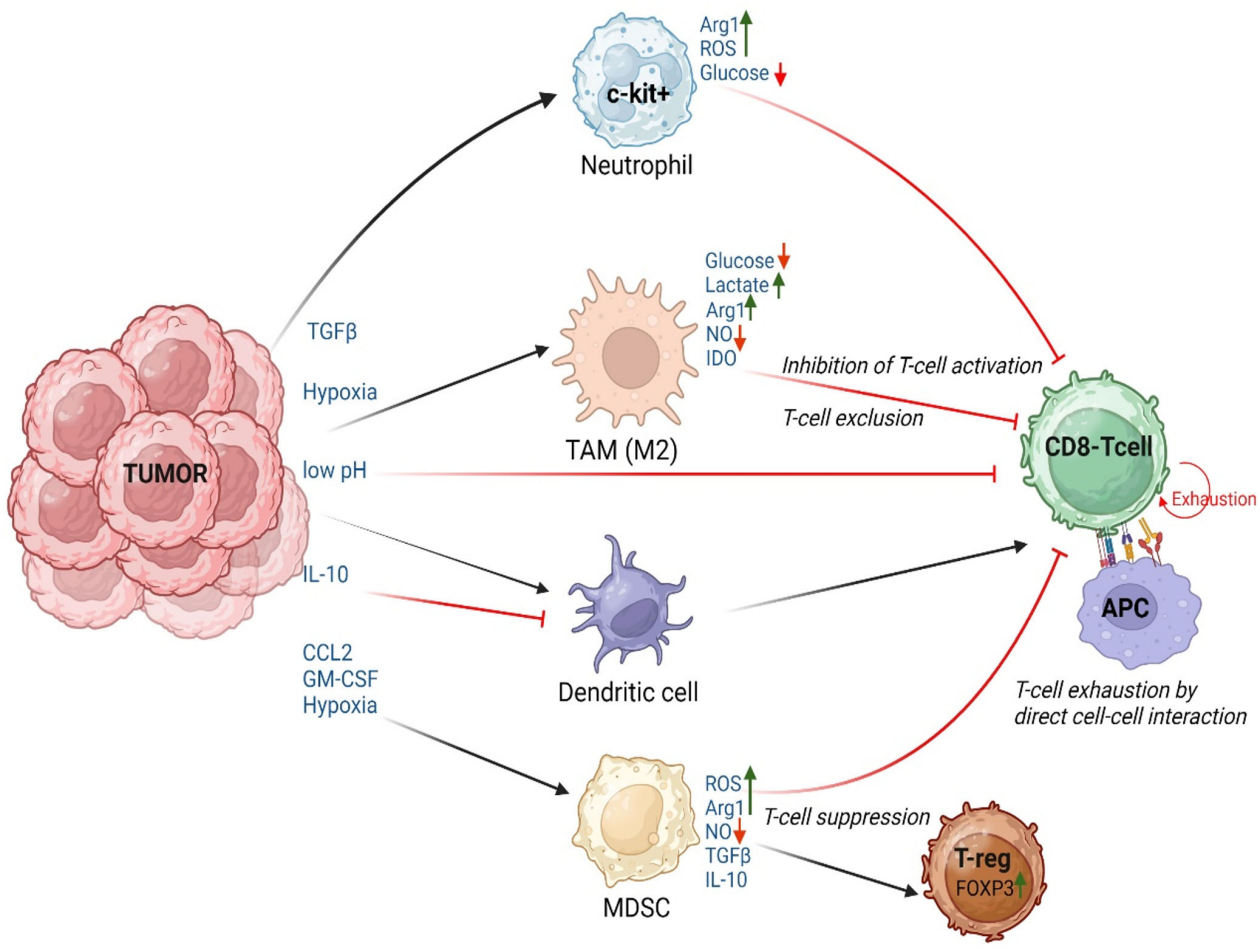
Innate cell mediated T-cell exhaustion in the TME: Schematic of the extracellular factors (pH, pO_2_), cytokines, chemokines, and metabolic changes within the TME that influence the innate cells to induce T-cell dysfunction by causing T-cell suppression/Differentiation into regulatory T-cells (T-reg), T-cell exhaustion due to direct cell-cell interactions or effects mediated by cytokines, T-cell exclusion, and inhibition of T-cell activation. APC, Antigen Presenting Cells, which could be Neutrophils, Dendritic cells, or monocytes/macrophages. Figure prepared using BioRender.

Innate cells dampen T-cell activity through several methods that include, T-cell suppression (by induction of Treg differentiation through TGF-β and IL-10), physical exclusion of tumor infiltrating lymphocytes (TILs) from the tumor core by TAMs/M2 macrophages (134), inhibition of T-cell proliferation and activation by limiting metabolites (135), and finally T-cell exhaustion. The current section of this review will focus on the mechanisms by which innate cells induce T-cell exhaustion within the TME either through direct T-cell-APC interactions (via binding of molecules such as PD-1, CTLA-4, LAG-3, Tim-3, TIGIT, B and T lymphocyte attenuator (BTLA), Glucocorticoid-induced tumor necrosis factor receptor-related protein (GITR) and CD160 to the respective binding partners on tumor cells or APCs) or indirectly through cytokine/chemokine alterations (such as reduction in levels of Granzyme B, TNF-α, IFN-γ, IL-2). Innate cells can also indirectly mediate T-cell exhaustion through metabolic switches that convert the differentiated, activated T-cells into an exhausted phenotype (**Figure 3**). These metabolic switches are often driven by epigenetic modifications in T-cells as detailed in section 4.2.

#### 5.2.1 Neutrophils

Increased tumor infiltration of neutrophils, often accompanied by formation of NETs, is known to promote tumor growth and metastasis (136, 137).

Recently, NETs have also been implicated as one of the factors paving the way for tumor immune escape by causing T-cell exhaustion in the TME. Tumor microenvironments rich in NETs were found to be populated by CD4 and CD8 T-cells that were metabolically and functionally exhausted. This could be due to the presence of high levels of PD-L1 within the NETs that directly interact with the T-cells (cell surface PD-1) and cause T-cell exhaustion (111).

#### 5.2.2 Dendritic cells

Dendritic cells have been classified into two main types: Conventional and Non-conventional. Conventional DCs (which are CD103^+^) present antigen via MHCI and MHCII to CD8^+^ and CD4^+^ T-cells, to induce a tumor cytolytic response or a Th1/Th2/Th17 response respectively (138). Non-conventional DCs, mainly myeloid-derived DCs (which are largely CD103^-^ and CX3CR1^+^) that are present within the inflammatory TME, produce TNFα and have increased expression of iNOS; and plasmacytoid DCs (largely CD303^+^ and CD123^+^) produce high levels of type 1 interferons upon sensing pathogen-associated (PAMPs) or damage-associated (DAMPs) molecular patterns (139, 140). Dendritic cells drive T-cell exhaustion primarily through CTLA-4 expressed on the surface of T-cells. Dendritic cells express both CD80 and CD86 that are ligands for CD28 and CTLA-4. CD86 is constitutively expressed while CD80 is expressed upon activation by soluble proteins such as IFN-γ, IFN-α, granulocyte-monocyte colony stimulating factor (GMCSF) present in a pro-inflammatory microenvironment and is repressed by IL-10 and IL-4 that are present in an anti-inflammatory/immunosuppressive state. CTLA-4 is transiently expressed on the surface of T-cells upon continuous stimulation and CD28-CD80/86 co-stimulation. CD28 is a high abundance, low affinity receptor for CD80/86 while CTLA-4 is a low abundance, high affinity receptor. Hence, when T-cells express both CD28 and CTLA-4, there is a possibility that DCs in the vicinity would preferentially bind to CTLA-4 and cause T-cell exhaustion (141).

#### 5.2.3 Myeloid-derived suppressor cells and tumor associated macrophages

MDSCs and TAMs are the immune suppressive cells present within the TME. These cells function in concert with one another and other non-immune cells such as the CAFs to suppress the activated T-cells within the TME mainly by modulating cytokine/chemokine balance as well as redox metabolism or induce infiltration/differentiation of regulatory T-cells. TAMs constitute the major fraction of immune cells within the TME of solid tumors (142). These TAMs express low levels of Nitric oxide synthase-2 (Nos2) and Cyclooxygenase-2 (Cox2), and high levels of Arginase-1 (Arg-1) and Indoleamine-2,3-dioxygenase (IDO) which maintains them in an immune suppressive M2 stage (143). Based on the type of stimuli present in the TME, M2 macrophages can exist as different subtypes (M2a, M2b, M2c or M2d) that release different chemokines and cytokines, and express distinctive cell surface markers (144). The type 2 cytokines IL-4 and IL-13 released by macrophages, MDSCs or even tumor cells, maintain the TAMs in an immune suppressed state (145). IL-4 has recently been found to cause exhaustion of CD8^+^ CAR T cells as well (146). IL-4 and IL-13, through the JAK/STAT pathway induce production of TGF-β which further maintains an immune suppressive TME. MDSCs also induce the differentiation of Tregs through TGF-β and IL-10 release and tip the redox balance in favor of a hypoxic, and acidic environment that impedes T-cell activity as discussed earlier in this review. TAMs use up the limited glucose supply in the TME via glycolysis and restrict the availability of glucose required for T-cell proliferation. Lactate, the byproduct of glycolysis acidifies the TME making it unfavorable for T-cell growth, eventually leading to T-cell exhaustion. A large share of the immune suppressive/T-cell exhaustive effects of MDSCs and TAMs, as well as some effects mediated by neutrophils in the TME, is because of the effects of these cells on T-cell metabolism. This is detailed in the following section.

## 6 Metabolic characteristics of exhausted T-cells and cross talk with innate-cell metabolism

The differentiation, proliferation, activity, and survival of T-cells are regulated by the availability of nutrients and their effective utilization. Different T cell states require specific metabolic programs to support their functions. Transitioning between these states involves active changes in cellular metabolism. For example, naïve T-cells maintain a quiescent state with minimal energetic and biosynthesis requirements and mainly derive energy through oxidative phosphorylation (OXPHOS) in the mitochondria (147, 148).

Differentiation of naïve T-cells into Teff requires generation of biomass and energy for rapid proliferation, which is supported by a shift from OXPHOS to aerobic glycolysis (149–151). Pentose phosphate pathway (PPP) which sustains biosynthesis and generates NADPH, is also enhanced to support effector functions (152). During transition from a naïve T-cell to a Teff, c-Myc, plays an important role in inducing the expression of glucose transporters (GLUT) through the activation of phosphoinositide 3-kinase (PI3K)/Akt and mTOR signaling pathways, thus enhancing glucose influx (153). In addition to carbohydrates, amino acid metabolism also has an indispensable role in T cell activation and effector differentiation (154, 155). Glutamine, tryptophan, and arginine are the three amino acids with well-described roles in Teff function. Glutamine metabolism allows ATP production in rapidly proliferating Teff and supports their development and functionality, by increasing IL-2 receptor expression and cytokine production (148). The enzyme IDO, catabolizes tryptophan to kynurenine and the high levels of toxic tryptophan-metabolites thus produced by mature APCs compromise Teff functions (156, 157). Concordantly, IDO inhibitors have been found to restore Teff function by preventing tryptophan breakdown within the TME (158, 158). Low arginine levels due to arginase activity of MDSCs and tumor cells impair aerobic glycolysis, Teff proliferation, reduce cytokine production and impair expression of activation markers such as CD25 and CD69. Besides carbohydrates and amino acids, Teff undertake *de novo* fatty acid synthesis to support lipid biosynthesis for building new membranes and generating signaling molecules (148).

Upon persistent antigen-exposure, T cells display poor effector function and become exhausted. The terminally exhausted T cells have impaired glycolysis and OXPHOS (159) accompanied by reduced glucose uptake and mitochondrial biogenesis. Terminally exhausted T cells express several immune co-inhibitory receptors. In this context, it has recently been reported that upon T-cell ligation, receipt of PD-1 signals can lower the capacity of T-cells to express GLUT1, subsequently leading to impaired glycolysis, and amino acid metabolism (160). PD-1 signaling is associated with reduced c-Myc expression, inhibition of activity of the PI3K/Akt/mTOR pathways and increase in β-oxidation of endogenous lipids (161). Expression of CTLA-4 and TIGIT also impair GLUT1 expression leading to induction of an exhausted T-cell phenotype (120, 162). Beyond overexpression of checkpoint receptors, lipid uptake and metabolism related enzymes are also induced in Tex. Among these enzymes, fatty acid translocase CD36, Acyl-CoA synthetase long chain family member 4 (ACSL4), cholesterol acyltransferase (ACAT), and 3-hydroxy-3-methyl glutaryl-coenzyme A reductase (HMGCR) have recently been reported to be related to T-cell exhaustion (163).

The metabolic demands of the different cell types present within the TME have a critical effect on T-cell activity and function. In addition to the tumor cells, macrophages are the major producers of lactate in the TME, thus playing a role in T-cell dysfunction (155). Macrophages can also modulate the availability of nutrients, such as glucose and amino acids, which are essential for T-cell metabolism (153). Within the hypoxic microenvironment of solid tumors, TAMs are particularly adept at fulfilling their energy requirements exclusively through glycolysis (144). Moreover, macrophages and MDSCs can produce kynurenine through IDO-mediated catabolism of tryptophan. Kynurenine pathway correlates with T-cell exhaustion in several cancers including melanoma (164, 165). MDSCs generate reactive oxygen species (ROS) and nitric oxide (NO), which can impair T-cell function by tipping the redox balance (166). Tumor neutrophils that are c-kit^+^ can also undergo oxidative metabolism in the glucose restricted TME, thereby further restricting glucose supply to T-cells while also generating ROS and keeping the TME immunosuppressed (167). Hence, combination of ICTs with metabolic intervention is expected to improve the effectiveness of immunotherapy by reversing T-cell exhaustion and dysfunction.

## 7 Molecular signatures of T-cell exhaustion

While considerable literature from studies on murine tissues has defined unique gene signatures for terminally exhausted T-cells, data from human tissue-derived Tex is limited. Recent independent studies have addressed this gap by conducting single-cell RNA sequencing (scRNA-seq) on patient-derived tissues to identify markers specific to Tex. To identify genes associated with Tex in human cancers, a PubMed search was conducted in September 2024 using the keywords “T-cell exhaustion and single-cell RNA sequencing in cancer,” yielding 232 results. From these, sixteen studies were identified that were published between 2016 and 2024, focusing on single-cell RNA sequencing (scRNA-Seq) conducted on patient-derived tumor samples. Data from human CD8^+^ T-cells derived from the tumor microenvironment revealed 158 genes upregulated in Tex compared to controls. **Table 2** consolidates 32 of these genes, that are common across at least four independent scRNA-seq studies.

**Table 2:** Upregulated genes associated with terminally exhausted T-cells (Tex) across multiple human tumor types.

	Tex genes
**Compared to**	Low exhausted CD8+ T-cells (168), non-exhausted cytotoxic CD8+ T-cells in tumor (169), Teff (170), Tn (171), Other clusters (172), Teff (173), Teff (174), Tn (175), Teff (176), Temra/Teff (177), T cells in low grade prostate cancer (178), CD8 T cells (179), Teff (180), Teff (181), Other clusters (182), Tn (183)
**Transmembrane proteins**	CTLA4 (62.5%), TIGIT (62.5%), HAVCR2 (62.5%), PDCD1 (56.25%), TNFRSF9 (43.75%), TNFRSF18 (37.5%), CXCR6 (37.5%), LAG3 (37.5%), NKG7 (31.25%), LAYN (31.25%), ENTPD1 (31.25%), TNFRSF1B (25%), CD27 (25%)
**Nuclear proteins/Transcription Factors**	CCND2 (31.25%), TOX (37.5%), RBPJ (25%), ZBED2 (25%), ZNF683 (25%)
**Cytokines, chemokines, granzymes**	CXCL13 (50%), IFNG (43.75%), GZMB (37.5%), CCL3 (37.5%), PRF1 (37.5%), FAM3C (31.25%), GZMA (25%), CSF1 (25%)
**Other genes**	ACP5 (25%), AKAP5 (25%), HLA-DMA (25%), KRT86 (25%), NDFIP2 (25%), SNAP47 (25%)

To identify genes associated with Tex in human cancers, a PubMed search was conducted in September 2024 using the keywords “T-cell exhaustion and single-cell RNA sequencing in cancer,” yielding 232 results. From these, sixteen studies were identified that were published between 2016 and 2024, focusing on single-cell RNA sequencing (scRNA-Seq) conducted on patient-derived tumor samples. Tumor types included are cervical cancer (n=8) (177)colorectal cancer (n=22) (173, 176) esophageal squamous cell carcinoma (n=8) (171) hepatocellular carcinoma (n=247) (169, 170, 173). lung squamous cell carcinoma (n=6) (174) lung adenocarcinoma (n=44) (175) metastatic melanoma (n=5) (168) non-small cell lung cancer (n=14) (173), ovarian cancer (n=8) (179, 181) pancreatic cancer (PAAD-CRA001160 dataset from the TISCH database with 190 datasets) (182), pan-cancer (21 cancer types, 316 samples) (172), low and high-grade prostate cancer (n=6) (178) thyroid carcinoma (n=23) (183), and urothelial bladder carcinoma (n=2) (180). The upregulated Tex genes compared to controls as mentioned in the table, were sourced as such from the text, figures, or supplementary tables of each study and compiled into a Microsoft Excel sheet. Conditional formatting was then applied to highlight duplicate genes across studies, and these duplicates were separately listed. A PivotTable in Microsoft Excel was used to count the frequency of each gene, and those identified in at least four of the sixteen studies (≥25% frequency) were included in this table. Genes were further classified based on their functional roles using the GeneCards and Reactome pathway databases, with categories including transmembrane proteins, nuclear proteins/transcription factors, chemokines, cytokines, granzymes, and other genes. All the values in parentheses following each gene name represent the percentage frequency across the 16 independent studies. Effector T cells (Teff), naïve T cells (Tn), and CD8+ T cells expressing CD45RA (Temra).

Consistent with murine studies, well-known Tex genes (CTLA4, LAG3, HAVCR2 (gene encoding Tim-3), PDCD1, TIGIT, TOX) were among the most prominently represented in Tex from human cancers. However, Tex in human cancers frequently expressed additional cell surface receptors (CD27, CXCR6, LAYN, NKG7, TNFRSF1B, TNFRSF9, TNFRSF18), transcription factors and nuclear proteins (CCND2, RBPJ, ZBED2, ZNF683), as well as chemokines, cytokines, and granzymes (CCL3, CSF1, CXCL13, IFNG, FAM3C, GZMA, GZMB, PRF1). These molecules may play a crucial role in regulating T-cell exhaustion in human cancers, as detailed in the next section.

### 7.1 Transmembrane receptors

Among the membrane markers of T-cell exhaustion, CD27, CD39, CXCR6, ENTPD1, LAYN, NKG7, TNFRSF1B, TNFRSF9, TNFRSF18 contribute to various aspects of cancer progression and T-cell exhaustion.

CD27, a lymphocyte-specific TNF receptor, plays a significant role through interaction with its ligand, CD70. In physiological conditions, this interaction generates a co-stimulatory signal for T-cell activation, whereas in cancers contributes to escaping anti-tumor immune surveillance by mediating apoptosis of T cells, driving TGF-β-mediated T cell exhaustion, and decreased apoptosis of Tregs (184, 185). Notably, agonistic anti-CD27 therapy, especially when combined with anti-PD-L1 treatment, has shown promising results. It boosts CD8^+^ T-cell proliferation, enhances cytokine production, and helps reverse quiescence in exhausted T-cells (184, 186, 187). These findings suggest that CD27-targeted therapies may be effective in revitalizing T-cells.

CXCR6, a receptor for the ligand CXCL16, is linked to cancer progression, promoting tumor growth and leukocyte migration. In breast cancer, CXCL16 was among the top myeloid-derived ligands to affect exhaustion-related target gene expression in CD8^+^ T-cells. However, whether CXCL16 acts through CXCR6 needs further investigation (188). It has been reported that exhausted CD8^+^PD1^+^ T cells accumulate in livers affected by non-alcoholic steatohepatitis (NASH). In preclinical models of NASH-related hepatocellular carcinoma, anti-PD1 treatment leads to more and larger tumor nodules, which are linked to an increase in CD8^+^PD1^+^CXCR6^+^TOX^+^TNF^+^ T cells (189). In metastatic melanoma, CXCR6 and TIM-3 co-expression on CD4^+^ T-cells may indicate pembrolizumab treatment failure (190). Additionally, findings from a pan-cancer study revealed predominant expression of CXCR6 in PD1^+^TIM3^+^CD8^+^ terminally dysfunctional T-cells promoting their survival through the expression of Tox, Bcl2, and CX3CR1 (191).

Ectonucleoside triphosphate diphosphohydrolase-1 (ENTPD1), also known as CD39, is an ectonucleotidase that generates adenosine, thereby suppressing the anti-tumor activities of CD4 and CD8 T-cells, as well as NK cells. Tumor upregulation of CD39 depletes immune-stimulatory extracellular ATP (eATP) in the tumor microenvironment, facilitating immune evasion and correlating with poor prognosis. CD39 also marks exhausted CD8^+^ TILs, which exhibit reduced cytokine production and cytotoxicity. In ovarian cancer, CD8^+^ T-cells co-expressing PD-1, TIGIT, CD39, and HLA-DR show signs of exhaustion. The blockade of CD39 has demonstrated potential to enhance T-cell proliferation and activation, indicating its therapeutic promise in reversing T-cell exhaustion (192, 193).

Layilin (LAYN), is a C-type lectin domain-containing glycoprotein. Although LAYN’s exact role in immunity is not well understood, recent studies indicate that LAYN may contribute to T cell exhaustion and suppress immune responses in the TME. Elevated levels of LAYN are associated with adverse survival outcomes in colorectal cancer, gastric cancer, and HPV-negative head and neck squamous cell carcinoma (HNSCC). In lung adenocarcinoma, therapies that reduce LAYN levels, such as combined anti-VEGFR2 and anti-PD1 treatments, have been shown to enhance anti-tumor immunity. Higher LAYN expression in HCC is also linked to poorer survival. Blocking LAYN can partially alleviate CD8^+^ T-cell exhaustion, highlighting its potential as a therapeutic target (194–198).

Natural killer cell granule protein-7 (NKG7) is a cytolytic granule-associated protein essential for the cytotoxic functions of immune cells. It is prominently expressed in NK cells, cytotoxic CD8^+^ T-cells, and cytotoxic CD4+ T-cells (199, 200). Although NKG7 is highly expressed in CD8^+^ effector T-cells, it is not crucial for CD8^+^ T-cell-mediated tumor control *in vivo* (200). Additionally, analysis of single-cell immune data from breast cancer studies identifies NKG7 as a CD8-Tex marker associated with significantly poorer survival outcomes (201).

Tumor Necrosis Factor Receptor Superfamily Member 1B (TNFRSF1B or TNFR2), one of the two receptors for tumor necrosis factor-alpha (TNFα), is involved in various immune and inflammatory responses. In ovarian cancer patients, the presence of an exhausted subpopulation of CD8^+^ TNFRSF1B^+^ T-cells is a marker of poor prognosis. Blockade of TNFRSF1B has been shown to reduce tumor growth by remodeling the immune microenvironment in mouse models of ovarian cancer (181).

Tumor necrosis factor receptor superfamily member 9 (TNFRSF9 or 4-1BB or CD137), a co-stimulatory receptor transiently expressed on activated T-cells and NK cells, has shown promise in activating CD8^+^ T-cells and eradicating tumors through agonist antibodies. However, its over expression is also associated with exhaustion markers such as PD-1, TIM-3, TIGIT, and CTLA-4. This dual role highlights the potential of TNFRSF9 as a target for reversing T-cell exhaustion and improving cancer therapy outcomes (202, 203).

Glucocorticoid-induced tumor necrosis factor receptor superfamily 18 (TNFRSF18), also known as GITR or CD357, is highly expressed on both Teff and Tregs (204). In CD8^+^ T-cells, GITR is essential for CD28-mediated costimulatory activity (204). However, single-cell RNA sequencing data from hepatocellular carcinoma (HCC) patients has identified TNFRSF18 as a key Tex gene associated with high-risk groups and poor patient survival (205). GITR is also overexpressed on functionally exhausted CD8^+^ TILs in microsatellite stable (MSS) colorectal cancer (CRC) (206). Notably, dual therapy combining GITR ligand (GITRL) with nivolumab (an anti-PD1 antibody) was shown to enhance the expansion of CD8^+^ TILs and improve the reinvigoration of IFN-γ secretion by exhausted CD8^+^ TILs from primary CRC (206).

### 7.2 Transcription factors

While TOX is a well-known key regulatory gene in Tex (207–211), other nuclear proteins and transcription factors have also been identified in these human single-cell RNA sequencing studies.

Zinc Finger BED-Type Containing 2 (ZBED2), a member of the ZBED gene family, encodes zinc finger domain-containing transcription factors involved in sequence-specific DNA binding. In pancreatic cancer, ZBED2 acts as an antagonist of IRF1 and, along with Recombination Signal Binding Protein for Immunoglobulin Kappa J Region (RBPJ) and ETS Variant Transcription Factor 1 (ETV1), is implicated in an exhausted T-cell phenotype (212).

Cyclin D2 (CCND2), a core component of cell cycle machinery, influences the growth and proliferation of cancer cells and is associated with tumorigenesis (213, 214). In non-small cell lung cancer patients, CCND2 levels were highly elevated in CD8^+^ T-cells undergoing exhaustion (215).

Recombination Signal Binding Protein for Immunoglobulin Kappa J Region (RBPJ) is an inhibitory transcription factor downstream of receptor activation in the Notch signaling pathway. High expression of RBPJ in HCC tissues is linked to poor prognosis and is positively correlated with various inhibitory receptors such as PDCD1, HAVCR2, CTLA4, LAG3, and TIGIT (216). The RBPJ–IRF1 axis has been shown to promote the differentiation of intermediate Tex into terminal Tex (217). Targeting RBPJ can enhance the functional and epigenetic reprogramming of Tex, thus improving therapeutic outcomes and efficacy of immune checkpoint blockade (ICB) therapy.

Zinc Finger Protein ZNF683/Hobit, a transcription factor homologous to Blimp1, is primarily expressed in effector T-cells, notably in quiescent and long-lived CD8^+^ T-cells (218). ZNF683/Hobit is a key regulator of the tissue-resident phenotype in T-cells (219). ZNF683/Hobit plays a crucial role in early stages of NK cell differentiation and may function to suppress IFN-γ production (220, 221). High ZNF683 expression has been observed in exhausted NK cells in nasopharyngeal carcinoma tissues (222).

### 7.3 Chemokines & cytokines

C-X-C Motif Chemokine 13 (CXCL13) plays a significant role in immune cell migration and tissue organization. In the context of lymph node metastasis, CXCL13-expressing exhausted CD8+ T-cells drive aggressive tumor phenotypes by enhancing interactions with tumor cells and activating ERK signaling pathways in tumor cells (223). Similarly, in gastric cancer, high levels of intra-tumoral CXCL13^+^CD8^+^ T-cells are linked to poor overall survival and reduced chemotherapy response, indicating T-cell exhaustion (224).

Macrophage Colony-Stimulating Factor 1 (CSF1) regulates macrophage differentiation and supports TAMs (225). In melanoma, CD8^+^ T-cells expressing CSF1 do not respond to PD1 checkpoint blockade. Preclinical studies suggest that combined inhibition of PD-1 and CSF1 receptor (CSF1R) can lead to tumor regression by depleting immunosuppressive and pro-tumorigenic TAMs, offering a strategy to counter CSF1-induced resistance (226). The CSF1/CSFR1 signaling pathway is critical for macrophage survival. Several FDA approved tyrosine kinase inhibitors such as Sorafenib and Sunitinib are known to target the CSF1/CSFR1 axis. Numerous clinical trials are currently underway to target CSFR1 to improve immunotherapy response (144).

FAM3C, a member of the Family with Sequence Similarity 3 (FAM3) cytokines, correlate strongly with LAG3 expression in dysfunctional CD8+ T-cells, hinting at their role in T-cell exhaustion (212).

CC Chemokine Ligand 3 (CCL3), also known as Macrophage Inflammatory Protein-1α (MIP-1α), binds to receptors CCR1 and CCR5, regulating dendritic cell homing and inducing antigen-specific T-cell responses in tumors (227). Conversely, CCL3 supports metastasis by recruiting cancer-associated fibroblasts (CAFs) via CCR5, leading to bone metastasis (228). Exhausted T-cells may secrete CCL3 to drive monocyte differentiation into suppressive TAMs that may further prime T cells toward exhaustion (229).

Granule associated enzyme A or Granzyme A (GZMA) is a serine protease identified in cytosolic granules (specialized secretory lysosomes or cytotoxic granules) of cytotoxic T and NK cells. Regarding its pro-tumorigenic role, mouse models of CRC, have demonstrated that pharmacological inhibition of extracellular GzmA reduces gut inflammation, prevents CRC development, and epithelial-to-mesenchymal transition (230). In breast cancer, GZMA correlates with T-cell checkpoints like PD-1, PD-L1, CTLA-4, TIGIT, and BTLA, however, its role in Tex is poorly understood (231).

Granzyme B (GZMB) is crucial in cytotoxic T lymphocytes and NK cells for killing infected and cancer cells. Beyond its cytotoxic role, GZMB may also exert pro-tumorigenic roles. GZMB mediated cleavage of its substrates in extracellular matrix such as decorin, fibronectin, vitronectin might facilitate tumor survival signaling and metastasis (232). High expression of GZMB in Tex and its functional impact on TME warrant further investigation.

Interferon Gamma (IFNG) is a cytokine with both pro- and anti-tumor effects, serving as a predictive marker for immunotherapy efficacy. Specific role of IFNG in Tex has not been deciphered. However, in support of its pro-tumorigenic function, it has been reported that IFN-γ promotes the expression of PD-L1, PD-L2 in tumor cells and other immune infiltrating cells. Interaction of PD-L1 or PD-L2 with an immune inhibitory receptor, PD-1 results in suppression of tumor-specific T cells or NK cells effector functions. Further IFN-γ enhances CTLA-4 expression on melanoma cells which causes immune evasion (233).

Perforin (PRF1), a cytotoxic protein in CTLs and NK cells, marks immune cells with killing capability and is central to the granule-dependent killing pathway (234). Tumors with high PRF1 expression show increased infiltration of CD8^+^ T-cells, CD4^+^ T-cells, NK cells, and M1 macrophages. However, high PRF1 levels also correlate strongly with exhausted T-cell markers (LAG-3, GZMB, PD-1, CTLA-4, TIM-3) (235).

Interestingly, high expression of GZMA, GZMB, IFNG, and PRF1 in exhausted T-cells may be attributed to feedback responses due to complex interactions with other stromal cells in the tumor microenvironment (TME). This interplay could contribute to immunosuppression, tumor progression and metastasis (236, 237). These insights highlight the complex roles of various chemokines, cytokines, and signaling modulators in T-cell exhaustion during tumor progression and suggest that targeting these molecules may offer new immunotherapy strategies.

## 8 Evidence for reversion of T-cell exhaustion and its mechanism

T-cell exhaustion occurs due to increased expression of immune checkpoint receptors and targeting these receptors (e.g., PD-1, LAG-3, TIM-3, CTLA-4, BTLA, TIGIT) may have the potential to reverse T-cell exhaustion. Currently, there are four types of immune checkpoint inhibitors (ICIs) approved by FDA for cancer treatment: (1) anti-CTLA-4 therapies: ipilimumab and tremelimumab (238, 239), (2) anti-PD-1 therapies: cemiplimab, dostarlimab, nivolumab, pembrolizumab, retifanlimab-dlwr and tislelizumab (240–245), (3) anti-PD-L1 therapies: atezolizumab, avelumab and durvalumab (246, 247), (4) anti-LAG-3 therapy: relatlimab (248). Other ICIs, such as TIM-3 and TIGIT inhibitors, are being evaluated in clinical trials. **Table 3** presents the clinical outcomes comparing single ICI treatments with combinations of ICIs. The premise of this table is to illustrate improved clinical response by combinatorial therapy on markers identified with T-cell exhaustion. It focuses on ICIs where single vs combinatorial therapy data is available. Key cancer indications were limited to melanoma, HNSCC, Non-Small Cell Lung Cancer (NSCLC), CRC, Renal Cell Carcinoma (RCC) and solid tumors. A pattern of improved clinical response represented as Objective Response Rates (ORR), Progression Free Survival (PFS) and Overall Survival (OS) can be observed with combination therapies across different indications, suggesting that targeting multiple checkpoint receptors leads to more effective clinical outcomes compared to single agent ICT. Reversion of exhausted T-cells to effector T-cells could be one of the reasons for improved clinical response to ICI combinations.

**Table 3:** Combinatorial immunotherapy potentially reinvigorates exhausted T-cells in solid cancers.

Target	Drugs	Status	Melanoma	HNSCC	NSCLC	CRC	RCC	Solid tumors	Key biomarkers
**CTLA4**	Ipilimumab	Approval	2011	Not approved					Low Lactate Dehydrogenase (LDH), Absolute Monocyte Counts (AMC), and MDSCs and high Absolute Eosinophil Counts (AEC), Tregs, and Relative Lymphocyte Counts (RLC) are associated with favorable outcomes following ipilimumab (249).
		Clinical response	mPFS 2.9 months. (NCT01844505) (250)	N/A	N/A	N/A	N/A	N/A	
**PD-1**	Nivolumab	Approval	2014	2016	2015	2017	2015	Not approved	Tumor PD-L1 expression is positively associated with response to anti-PD-1 blockade. First FDA approved as predictive biomarker for Pembrolizumab treatment of NSCLC in 2015, it is widely used as predictive biomarker for anti-PD1 blocking ICIs (251).
		Clinical response	mPFS 6.9 months. (NCT01844505) (250)	ORR 18.3%. (NCT02823574) (252)	Nivo+chemo: mEFS 31.6 months (NCT02998528) (253)	ORR 31% (at 12 months). (NCT02060188) (254)	mOS 25.8 months, mPFS 4.2months, ORR 23% (NCT01668784) (255)	N/A	
**PD-1**	Pembrolizumab	Approval	2014	2016	2015	2020	2021	2017	Microsatellite Instability/Deficient Mismatch Repair (MSI/dMMR) and TMB are other FDA-approved predictive biomarkers for the pembrolizumab treatment of solid tumors (256).
		Clinical response	Survival rate 74.1% (at 12 months) (NCT01866319) (257)	OS 38% (at 12 months) (NCT01848834) (258)	mOS 22.1 months in patients with PD-L1 Tumor Proportion Score (TPS) ≥50% (NCT01295827) (259)	mPFS 16.5 months (NCT02563002) (260)	DFS was better with pembrolizumab compared with placebo (at 24 months, 77.3% versus 68.1%) (NCT03142334) (261)	ORR 29% (NCT02628067) (262)	
**CTLA4 + PD-1**	Ipilumumab + Nivolumab	Approval	2015	Not approved	2020	2018	2018	Not approved	In melanoma, PD-L1-positive patients had mPFS of 14.0 months in both the nivolumab plus ipilimumab and nivolumab alone groups, but in PDL1-negative patients, mPFS was longer with the combination as compared with nivolumab alone (11.2 months versus 5.3 months), suggesting PD-L1 negative patients will benefit more with the combination treatment (263). In NSCLC, Nivolumab plus ipilimumab was seen to be effective, regardless of PD-L1 expression (264). In CRC, DNA Mismatch Repair–Deficient/Microsatellite Instability–High Metastatic Colorectal Cancer patients benefit from the treatment (NCT04008030). TMB is a key predictive biomarker for immunotherapy spanning a range of solid tumor types. In patients with advanced or metastatic tumor mutational burden (TMB)–high solid tumors that were refractory to standard therapies, combination treatment showed promise (265).
		Clinical response	Combination: mPFS 11.5 months, vs single Ipilimumab: mPFS 2.9 months, Nivolumab: mPFS 6.9 months. (NCT01844505) (250)	Population with platinum-refractory disease Combination: ORR 13.2%, vs single Nivolumab: ORR 18.3%. Population with platinum-eligible disease Combination: ORR 20.3%, vs single Nivolumab: ORR 29.5% (NCT02823574) (252)	Combination: 5yr OS 24% (>=1% PDL1), Combination: 5yr OS 19% (<1% PDL1). (NCT02477826) vs single Nivo+chemo: mEFS 31.6 months (NCT02998528) (266)	Combination: ORR 55%, vs single Nivolumab: ORR 31%. (NCT02060188) (254) mPFS was not reached with nivolumab plus ipilimumab and was 39.3 months with nivolumab (NCT04008030)	Combo: ORR 61% (NCT02231749; 5 year data) vs single ORR 23% (NCT01668784) (255)	Combo: ORR 38.6% (tTMB-H), ORR 22.5% (bTMB-H). vs single Nivolumab: ORR 29.8% (tTMB-H) and 15.6% ((bTMB-H). (NCT03668119) (265)	
	Ipilumumab + Pembrolizumab	Approval	Not approved						In melanoma, more responses were observed in PD-L1–negative archival tumors (15 of 39 [38%]) as compared with PD-L1–positive archival tumors(4 of 27 [15%]). PD-L1 negative patients will benefit more with the combination treatment and can be considered as a potential biomarker. NCT02743819.
		Clinical response	Combination following Anti-PD-1/L1 Failure: mPFS 5.0 months, and the mOS 24.7 months. (NCT02743819) (267)	N/A	Combination: mOS 21.4 months vs single Pembrolizumab: mOS 21.9 months (NCT03302234) (268)	N/A	N/A	N/A	
**LAG-3 + PD1**	Opdualag (nivolumab and relatlimab-rmbw)	Approval	2022	Not approved					In melanoma, patients with ≥ 1% LAG-3+ cells in their tumors had significantly longer PFS compared to patients with < 1% LAG-3 expression. No significant difference was observed in overall survival between the two groups (269).Further evaluation of LAG-3 and PD-L1 to determine their roles as predictive biomarkers for response to Opdualag might be beneficial.
		Clinical response	RELA + NIVO: OS 63.7% (at 24 months), vs single Nivo: OS 58.3% (at 24 months) (NCT03470922)	Phase II: NCT04080804, NCT04326257	Phase II: NCT04205552, NCT04623775	Phase III: NCT05328908 PhaseII: NCT03642067	N/A	PhaseII: NCT04095208, NCT03607890, NCT05134948	
	Eftilagimod alpha + Pembrolizumab	Approval	Not approved						In HNSCC, better response was seen in PD-L1 subgroup of patients (PD-L1 Combined positive score (CPS) ≥20), 12-month OS rate 66.7% vs 46.0% in unselected for PD-L1 and ORR 60% vs 30% in unselected for PD-L1 (NCT03625323). In NSCLC, the combination of efti, pembrolizumab, and chemotherapy led to an ORR of 55.0%. Among those with a PD-L1 tumor proportion score (TPS) of more than 50%, the ORR was 75.0%. In patients with a PD-L1 TPS of 1% to 49%, the ORR was 58.8%. Additionally, the ORR was 47.4% among those with a PD-L1 TPS of less than 1% (NCT03252938). PD-L1 can be a positive predictive biomarker for this combination treatment, however, further evaluation is warranted.
		Clinical response	N/A	12-month OS rate 46.0%. The ORR 30% for second-line treatment of 37 patients with HNSCC (NCT03625323) and Phase II: NCT04811027. vs single Pembrolizumab OS 38% (at 12 months) (NCT01848834) (258)	2022 (fasttrack designation). Minimum follow-up of 22 months (n = 21), combination plus chemotherapy mOS 32.9 months and a 24-month OS rate of 81.0%. (NCT03252938) vs single median follow-up of 23.1 months, pembrolizumab plus chemotherapy alone (n = 410), mOS of 22.0 months and a mPFS of 9.0 (NCT02578680)	N/A	N/A	N/A	
**TIGIT+PD1/PD-L1**	Vibostolimab +Pembrolizumab	Approval	Not approved						In NSCLC naive to PD-(L)1 inhibitors, ORR was 33% in the PD-L1 tumor proportion score (TPS) ≥1% subgroup and 27% in the PD-L1 TPS < 1% subgroup. Preliminary data suggests the use of PD-L1 as a predictive therapeutic biomarker, however, more data is needed (270). In patients with PD-L1–positive HNSCC, PD-L1 and TMB showed trends of a positive association with response; TIGIT, Tcell_inf_GEP, and *PVR* did not show an association with response (NCT05007106) (270).
		Clinical response	N/A	N/A	In NSCLC naive to PD-(L)1 inhibitors, ORR: 26% with sequentially administered vibostolimab plus pembrolizumab. In patients with anti-PD-1/PD-L1-refractory NSCLC ORR 3% vs single Vibostolimab (anti-TIGIT) ORR 3% (NCT02964013) (271)	N/A	N/A	ORR 7% vs single Vibostolimab (anti-TIGIT) ORR 0% (NCT02964013) (271)	
	Tiragolumab+ Atezolizumab (anti PD-L1)	Approval	Not approved						Studies suggest that PD-L1 expression, may be a biomarker for tira + atezo combination therapy in metastatic PD-L1-positive untreated NSCLC. (NCT03563716)
		Clinical response	N/A	N/A	ORR 37.3% in combination vs single 20.6% in placebo+ Atezolizumab (NCT03563716)	N/A	N/A	N/A	

Tabulated representation of clinical outcomes comparing single treatments (targeting CTLA4/PD1/PD-L1) with combination treatments (targeting CTLA4 with PD1, LAG3 with PD1, TIGIT with PD1/PD-L1), key cellular receptors now associated with T-cell exhaustion. The information in this table is derived from completed or ongoing clinical trials from Clinicaltrials.gov with published clinical response data for combination treatments using Pubmed, abstracts published in ASCO and other online journals. Clinical outcomes were reported as Objective Response Rates (ORR), median Progression Free Survival (mPFS), median Overall Survival (mOS), median Event Free Survival (mEFS), Tumor Proportion Score (TPS), Disease-Free Survival (DFS), Complete Response (CR), Partial Response (PR) from Clinical trials PhaseI/II/III (as indicated in the table; Phase I data alone is included when PhaseII/III is not available). Since there are several ongoing clinical trials across multiple cancers, key cancer indications were limited to melanoma, Head and Neck Squamous cell Carcinoma (HNSCC), Non-Small Cell Lung Cancer (NSCLC), Colorectal Cancer (CRC), Renal Cell Carcinoma (RCC) and solid tumors (in general), therefore this table will not contain information from all ongoing or completed clinical trials for these and other indications. The table is an update of the combinatorial effect of ICIs compiled from publications, abstracts, or online reports, spanning over ten years, from Ipilimumab (approved in 2011) to Opdualag (combination of LAG-3 and PD1 inhibition, approved in 2022 for metastatic melanoma). This table illustrates improved clinical response observed in trials using combinations of ICBs developed against the various markers of T-cell exhaustion compared to single agent ICT. N/A: Data not available.

Apart from the trials mentioned in the table, there are several ongoing early phase trials, for which clinical response data is currently not available. However, preclinical studies supporting these clinical trials suggest potential efficacy. For example, in tumor bearing mice models, TIGIT^+^ NK cells acquired an exhaustion phenotype, with reduced effector function and antitumor potential. Lack of TIGIT expression or blocking of TIGIT specifically in NK cells using antibodies, retarded tumor growth and reversed antitumor NK cell exhaustion (272). TIGIT is being investigated in combination with FDA approved ICIs in clinical trials for NSCLC and solid tumors (**Table 3**).

Tim-3 was found to mark the most exhausted subset among tumor infiltrating CD8^+^PD-1^+^ T-cells and co-targeting of Tim-3 and PD-1 pathways-controlled tumor growth (47, 272). It has been shown that Tim-3 expression also associates with NK cell exhaustion and disease progression in patients with melanoma, lung adenocarcinoma, HCC, and esophageal cancer. In melanoma and lung adenocarcinoma, exhausted NK cell phenotype could be reversed by Tim-3 blockade (273–276). Anti-TIM-3 antibody, Cobolimab plus dostarlimab (anti-PD-1) has shown promise in advanced hepatocellular carcinoma (NCT03680508). Tim-3 monoclonal antibody, LY3321367, singly or in combination with the anti-PD-L1 antibody, LY300054 are being assessed in clinical trials, for patients with advanced solid tumors (277). The anti-TIM-3 antibody, Sabatolimab (MBG453) along with spartalizumab (anti-PD-1 antibody) has shown early signs of efficacy in multiple solid tumors (278) Several other TIM-3 targeting therapeutics are also being assessed in early phase clinical trials, alone or in combination with other ICIs (279)=].

In ovarian cancer patients, LAG-3 and PD-1 co-expression is associated with the dysfunction or depletion of CD8^+^ T-cells, suggesting that co-inhibition of these two receptors can help revive these cells from the exhausted state (280). A combination of relatlimab and nivolumab (Opdualag™) was approved by FDA in 2022, for treatment of unresectable or metastatic melanoma (**Table 3**). A triple combination involving Opdualag™, and the CTLA4 inhibitor Yervoy is being tried for treatment of advanced melanoma and initial results after 49.4-month median follow-up of a cohort in the phase 1/2 RELATIVITY-048 (NCT03459222) trial show confirmed ORR of 58.7% and a 48-month OS rate of 69.1%. (Meeting abstract 9504, 2024 ASCO Annual Meeting I). A PD-1/LAG-3 bispecific antibody, RO7247669/Tobemstomig is in early Phase I/II clinical trial for use in patients with melanoma, NSCLC, RCC and solid tumors (NCT05116202, NCT05419388, NCT05775289, NCT05805501, NCT04140500). Another humanized anti–LAG-3 monoclonal antibody (Favezelimab/MK-4280), in combination with pembrolizumab (Keytruda) is currently in clinical trials for treatment of melanoma, NSCLC, CRC and RCC (trial numbers: NCT04305054, NCT04303169, NCT04938817, NCT03516981, NCT05600309, NCT05064059, NCT04895722, NCT04626518, NCT04626479). A phase I trial of Fianlimab (LAG-3 inhibitor) in combination with cemiplimab in melanoma patients showed favorable response (NCT03005782) (281). Currently, several Phase II/III trials are ongoing to test this combination therapy for melanoma, HNSCC and NSCLC (trial numbers: NCT05608291, NCT05352672, NCT03916627, NCT05785767, NCT058000, Meeting abstract 9548, 2023 ASCO Annual Meeting I).

## 9 Discussion

While exhausted T-cells are identified as markers of an ineffective immune response, they play a nuanced role in cancer and for controlling infections. Depletion of CD8+T exhausted cells leads to reduced control of tumor growth in pre-clinical animal models. Depletion of Tex cells improved viral replication during chronic LCMV infection in mice or Simian Immunodeficiency Virus infection in rhesus macaques (282–285). The fact that single agent ICT or combinations of different ICBs targeting either receptors or ligands of T-cell inhibition pathways, can re-invigorate exhausted CD8^+^ T-cells by enhancing their anti-viral (286) or anti-tumor activity in various animal models and in humans (287) would suggest that not all exhausted CD8^+^ T-cells are terminally differentiated or dysfunctional. Some cells are still capable of sustaining a CD8^+^ T-cell response and conferring protection. Recently (282, 288), the authors in their opinion pieces have argued that “functional adaptation” would be a more appropriate term for T-cell “exhaustion” as CD8^+^ T-cells may continuously adjust their differentiation states in response to sustained antigen exposure to better meet the needs of the body during a chronic infection by maintaining viral control without causing overwhelming immunopathology and the same would apply to cancers as well. A body of recent literature suggests that while the terminally differentiated Tex cells may be too far along in the differentiation continuum to revert to Teff phenotype given their fixed epigenetic state, this may not be true for the progenitor and intermediary Tex cells identified by expression of the transcription factors TCF-1 and TOX. Studies discussed in this review contend that understanding the differentiation pathway of Tex in solid tumors will facilitate identification of druggable targets for better anti-tumor efficacy and for developing better therapeutic regimen to revert Tex to Teff within the TME of solid tumors.

In this review (Section 7), curation of publicly available single-cell RNA sequencing data across human cancers, identified key genes (such as surface receptors, transcription factors, nuclear proteins, chemokines and cytokines) associated with human T-cell exhaustion (**Table 2**). It is interesting to note that some of these genes such as CD27, ENTPD1 (CD39), TNFRSF9 (CD137), TNFRSF18 (GITR), and CSF1/CSF1R are being actively targeted in ongoing clinical trials. Most of them are in combination with established immune checkpoint inhibitors like PD-1, LAG-3, and CTLA-4. For example, agonistic anti-CD27 antibodies, when combined with anti-PD-L1 therapies, disrupt quiescence in exhausted CD8^+^ T cells, enhancing their proliferation and effector cytokine production (187). A Phase 1/2 study of varlilumab (anti-CD27) with nivolumab demonstrated good tolerance and clinical activity, particularly in ovarian cancer (289). CD27 co-stimulation is also crucial for boosting CAR-T cell anti-tumor efficacy (290). ENTPD1 (CD39), a marker of T cell exhaustion, has been demonstrated to be targeted with nanobody constructs to increase T cell proliferation and activation (193). Clinical trials are investigating anti-CD39 strategies with PD-1 blockade and chemotherapy (291–296). Anti-CD39 therapy may increase the proliferation of tumor infiltrating lymphocytes, while anti-PD-1 may reverse the exhaustion phenotype of these lymphocytes. Together, these treatments may achieve a synergistic antitumor effect. CD137 (TNFRSF9) is another promising target. Agonistic antibodies against CD137 have shown potential in enhancing T cell activation, memory, and effector function, with studies demonstrating improved survival in pancreatic cancer models (297, 298). ATOR-1017, a CD137 agonist, showed promising clinical activity and safety in Phase I trials (299, 300). Similarly, a first-in-human study of utomilumab (CD137 agonist) combined with rituximab demonstrated clinical activity and safety in CD20+ NHL patients (301). GITR (TNFRSF18) stimulation can reinvigorate exhausted tumor-infiltrating lymphocytes, particularly when combined with anti-PD-1 therapy. This combination has shown potential in colorectal cancer (206). A Phase I/Ib study of GWN323 (anti-GITR) with spartalizumab (anti-PD-1) showed tolerability and modest clinical benefit in advanced solid tumors (302). Ongoing studies continue to investigate this combination in advanced tumors (NCT02628574 (303),). Finally, CSF1/CSF1R-targeting agents have demonstrated a favorable safety profile and potent blocking activity, particularly in diffuse-type tenosynovial giant cell tumors (TGCT) (304). FF-10101, a CSF1R inhibitor, enhanced antitumor effects when combined with anti-PD-1 therapy (305). Vimseltinib, another CSF1R inhibitor, showed long-term tolerability and antitumor activity in TGCT patients not amenable to surgery (306). Other immune-modulatory molecules like LAYN and RBPJ (198, 216) hold untapped potential to reinvigorate exhausted T cells and significantly improve therapeutic outcomes. While these examples suggest potential therapeutic options for patients, additional genes listed in section 7 may be useful for novel therapeutic approaches either with existing ICT combination or other innovative therapeutic strategies.

Despite the significant treatment advantage recently imparted by ICT, the response rate is low for many cancers. This initial resistance to ICT is majorly due to a general dearth of tumor T-cell infiltration (characteristic feature of a ‘cold tumor’) (307). The paucity of T-cells within the TME is due to several reasons such as lack of tumor antigens, defective antigen presentation, absence of T-cell activation, exclusion of T-cells from the tumor niche/tumor bed (307). Regulating each of these events using different therapies should in theory be able to convert a cold tumor to a hot tumor. Several strategies involving the combination of immunotherapy with conventional chemotherapy and radiotherapy, as well as epigenetic modifiers and multifunctional antibody therapeutics are now being developed to promote this switch. The conversion of a cold tumor to a hot tumor potentially paves the way for preventing or reverting T-cell exhaustion and improving immunotherapy response.

A case study report of a metastatic pancreatic cancer patient revealed that a combination of chemotherapy (oxaliplatin) with immunotherapy and fractional radiation treatment was able to change the features of the TME to a “hot tumor” phenotype via several methods including activation of cGAS-STING, increased CXCL9 and CXCL10 leading to improved CD8+T-cell infiltration and also by inhibiting DNA damage repair (308). Stereotactic body radiotherapy (SBRT) in combination with immunotherapy has shown to stimulate local and systemic antitumor immune responses in addition to direct tumor killing in advanced NSCLC patients (309). It has recently been found that certain chemotherapeutics such as doxorubucin, paclitaxel and oxaliplatin can induce immunogenic tumor cell death via release of Pathogen-associated molecular patterns (PAMPs) and Damage-associated molecular patterns (DAMPs) which then enhance DC maturation and antigen presentation followed by improved tumor T-cell infiltration. This process called “inducing chemotherapy” is where chemotherapy is given prior to immunotherapy to make the tumor hot and hence improve the efficacy of the immunotherapy that follows. This is particularly observed and relevant in CRC (trials ongoing) which is difficult to treat and has a mOS of 30 months (310).

Bifunctional or bispecific or multi-specific antibodies have tumor targeting and immune activating arms. This could be T-cell engagers or cytokines or cytokine receptors to name a few. In this scenario, such therapeutics may be able to convert any cold tumor to a hot tumor. For example, previous studies from our group indicate that in combination with anti-PD1 and ficerafusp alfa (BCA101), an anti-EGFR with a TGF-β trapping arm was able to significantly reduce the growth of tumors produced by B16-hEGFR (B16 cells engineered to overexpress human EGFR) (311), an otherwise cold syngeneic tumor model.

Other strategies to fine-tune anti-tumor immune responses include development of novel multifunctional molecules, optimizing cytokine signaling pathways, epigenetic modifiers, and modulating mitochondrial metabolism, as well as targeting metabolic checkpoints and co-stimulatory molecules.

Continued research and clinical trials are essential to validate these targets and optimize therapies and combinations, ultimately improving patient outcomes and advancing the fight against cancer. Immunotherapies and their combinations must be used cautiously as an overzealous Teff response results in immunopathology (286). Treatments aimed at reversing T cell exhaustion can lead to overactive T cell responses, resulting in Cytokine Release Syndrome (CRS), chronic inflammation of organs, and autoimmune conditions like colitis, dermatitis, thyroiditis and other related disorders (312, 313). Several therapeutic options such as corticosteroids, infliximab (anti-TNF antibody) and tocilizumab (anti-IL-6 antibody) can be effective in managing CRS (312, 313). While immune checkpoint combinatorial therapies offer promising benefits by reversing T cell exhaustion and improving anti-tumor response, it is essential to minimize the risk of immune related Adverse Events (irAEs) in patients. Therefore, patient-specific management is crucial, as immune-related adverse events can vary significantly between individuals. Balancing clinical efficacy with safety will be key for the next generation of therapeutics targeting exhausted T-cells within the tumor microenvironment.
